# Altered Expression of Genes Encoding Neurotransmitter Receptors in GnRH Neurons of Proestrous Mice

**DOI:** 10.3389/fncel.2016.00230

**Published:** 2016-10-07

**Authors:** Csaba Vastagh, Annie Rodolosse, Norbert Solymosi, Zsolt Liposits

**Affiliations:** ^1^Laboratory of Endocrine Neurobiology, Institute of Experimental Medicine, Hungarian Academy of SciencesBudapest, Hungary; ^2^Functional Genomics Core, Institute for Research in Biomedicine (IRB Barcelona)Barcelona, Spain; ^3^Department of Animal Hygiene, Herd-Health and Veterinary Ethology, University of Veterinary MedicineBudapest, Hungary; ^4^Department of Neuroscience, Faculty of Information Technology and Bionics, Pázmány Péter Catholic UniversityBudapest, Hungary

**Keywords:** GnRH neuron, gene expression, proestrus, neurotransmission, genomics, microarray analysis, pathway analysis, mouse

## Abstract

Gonadotropin-releasing hormone (GnRH) neurons play a key role in the central regulation of reproduction. In proestrous female mice, estradiol triggers the pre-ovulatory GnRH surge, however, its impact on the expression of neurotransmitter receptor genes in GnRH neurons has not been explored yet. We hypothesized that proestrus is accompanied by substantial changes in the expression profile of genes coding for neurotransmitter receptors in GnRH neurons. We compared the transcriptome of GnRH neurons obtained from intact, proestrous, and metestrous female GnRH-GFP transgenic mice, respectively. About 1500 individual GnRH neurons were sampled from both groups and their transcriptome was analyzed using microarray hybridization and real-time PCR. In this study, changes in mRNA expression of genes involved in neurotransmitter signaling were investigated. Differential gene expression was most apparent in GABA-ergic (*Gabbr1, Gabra3, Gabrb3, Gabrb2, Gabrg2*), glutamatergic (*Gria1, Gria2, Grin1, Grin3a, Grm1, Slc17a6*), cholinergic (*Chrnb2, Chrm4*) and dopaminergic (*Drd3, Drd4*), adrenergic (*Adra1b, Adra2a, Adra2c*), adenosinergic (*Adora2a, Adora2b*), glycinergic (*Glra*), purinergic (*P2rx7*), and serotonergic (*Htr1b*) receptors. In concert with these events, expression of genes in the signaling pathways downstream to the receptors, i.e., G-proteins (*Gnai1, Gnai2, Gnas*), adenylate-cyclases (*Adcy3, Adcy5*), protein kinase A (*Prkaca, Prkacb*) protein kinase C (*Prkca*) and certain transporters (*Slc1a4, Slc17a6, Slc6a17*) were also changed. The marked differences found in the expression of genes involved in neurotransmitter signaling of GnRH neurons at pro- and metestrous stages of the ovarian cycle indicate the differential contribution of these neurotransmitter systems to the induction of the pre-ovulatory GnRH surge, the known prerequisite of the subsequent hormonal cascade inducing ovulation.

## Introduction

Gonadotropin-releasing hormone (GnRH) neurons play a fundamental role in the maintenance of reproduction (Knobil and Neill, 2006). GnRH axons project to the median eminence where they release (Merchenthaler et al., 1980) GnRH into the portal circulation to regulate the pituitary-gonadal axis (Carmel et al., 1976). GnRH neurons are controlled by different neuronal networks via specific membrane receptors for neurotransmitters and neuropeptides released from the presynaptic neuronal afferents (Smith and Jennes, 2001; Campbell, 2007) that modulate the synthesis rate and release pattern of GnRH and other co-produced neuromodulators (Finn et al., 1998; Christian and Moenter, 2010). The operation of the hypothalamo-pituitary gonadal (HPG) axis is cyclic including the cellular activity of GnRH neurons (Plant, 2015). Gonadal hormones have a substantial role in the modulation of GnRH neurons and their neuronal afferents (Radovick et al., 2012).

In female rodents, estradiol (E2) exerts biphasic effects on GnRH neurons and the release of the decapeptide (Sarkar and Fink, 1980; Herbison, 1998). It predominantly suppresses the GnRH system via negative feedback effects with the exception of proestrus when the rising level of E2 primes the system for the preovulatory GnRH surge (Sarkar et al., 1976). This process is driven by E2 acting on estrogen receptors (ERα, ERβ, GPR30 and STX-sensitive membrane receptors; Chu et al., 2009; Terasawa et al., 2009; Kenealy et al., 2011; Moenter and Chu, 2012). While the afferent systems of GnRH neurons are known to be regulated predominantly by ERα (Wintermantel et al., 2006; Christian et al., 2008; Yeo and Herbison, 2014; Cheong et al., 2015), GnRH neurons express exclusively the beta subtype of the nuclear receptor (Hrabovszky et al., 2000, 2001). The positive E2 feedback, therefore, can target the widespread neuronal regulators of the GnRH system and also the GnRH neurons themselves via direct actions. The neuronal networks mediating the negative and positive feedback effects of E2 to GnRH neurons have been extensively studied by morphological and functional tools (Wintermantel et al., 2006; Christian et al., 2008; Yeo and Herbison, 2014). In the preovulatory GnRH surge period, GnRH neurons undergo activation exemplified by expression of the immediate early gene c-Fos (Lee et al., 1990), increased transcriptional activity (Chiu et al., 1988; Wang et al., 1995), induction of hormone synthesis (Gore and Roberts, 1997; Finn et al., 1998) and altered firing pattern (Christian et al., 2005; Farkas et al., 2013).

The classical neurotransmitter systems of the brain are potent regulators of the GnRH system as reviewed earlier (Smith and Jennes, 2001). Powerful regulatory role has been revealed for gamma-aminobutyric acid (GABA) (Herbison and Moenter, 2011), glutamate (Iremonger et al., 2010) dopamine (DA) (Liu and Herbison, 2013), norepinephrine (NE) (Hosny and Jennes, 1998), serotonin (Bhattarai et al., 2014), acetylcholine (ACh) (Turi et al., 2008), and histamine (H) (Fekete et al., 1999). In concert with the rich communication of GnRH neurons with diverse transmitter systems of the brain, the expression of genes encoding for neurotransmitter receptors (Todman et al., 2005) and ion channels (Bosch et al., 2013; Norberg et al., 2013) in GnRH neurons has also been verified. In the present study, the proestrus-associated changes in the expression of neurotransmitter receptor genes of GnRH neurons have been challenged. To achieve this goal, we carried out microarray- and PCR-based transcriptome analysis of GnRH neurons harvested from regularly cycling GnRH-GFP transgenic mice at proestrus and metestrus stages of the ovarian cycle. The comparative study revealed a differential expression of neurotransmitter receptor genes of GnRH neurons in proestrous mice providing novel data to have a better understanding of the communication and plasticity between GnRH neurons and their afferents under the positive feedback action of estradiol.

## Materials and methods

### Ethics statement

All experiments were performed with permissions from the Animal Welfare Committee of the Institute of Experimental Medicine Hungarian Academy of Sciences (Permission Number: A5769-01) and in accordance with legal requirements of the European Community (Decree86/609/EEC). All animal experimentation described was conducted in accordance with accepted standards of humane animal care and all efforts were made to minimize suffering.

### Animals

Adult, gonadally intact female mice were used from local colonies bred at the Medical Gene Technology Unit of the Institute of Experimental Medicine (IEM). They were housed in light (12:12 light-dark cycle, lights on at 06:00 h)—and temperature (22 ± 2°C) controlled environment, with free access to standard food and tap water. GnRH-green-fluorescent protein (GnRH-GFP) transgenic mice (Suter et al., 2000) bred on a C57BL/6J genetic background were used. In this animal model, a GnRH promoter segment drives selective GFP expression in the majority of GnRH neurons. The estrous cycle was monitored daily between 9 and 10 a.m. by microscopic evaluation of vaginal cytology (Nelson et al., 1982; Byers et al., 2012; Cora et al., 2015). Proestrous (*n* = 6) and metestrous (*n* = 6) female mice with at least two consecutive, regular estrous cycles were used. In order to avoid the possible circadian effect, animals were sacrificed at the same period of the day, between 16:00 and 18:00 h. Those animals were considered to be in the proestrus stage that fulfilled the following criteria: (1) vaginal smear staining with predominance of nucleated epithelial cells (Byers et al., 2012); (2) LH serum concentrations >5 mg/L (15.11 ± 3.4 mg/L); (3) uterus wet weights >0.15 g (0.19 ± 0.01 g). Accordingly, the following criteria were applied for the metestrous cycle phase: (1) vaginal smears consisting of the three cell types: leukocytes, cornified and nucleated epithelial cells (Byers et al., 2012); (2) serum LH levels < 0.5 mg/L (0.35 ± 0.02 mg/L); (3) uterus wet weights < 0.1 g (0.08 ± 0.01 g). Serum LH concentrations and uterus weight data are presented in Supplementary Figure 1.

### Serum LH measurements

Blood samples were collected from the heart of deeply anesthetized mice immediately before the brain fixation step. The samples were chilled on ice, centrifuged at 1300 g for 3 min at 4°C. Plasma was aspirated then frozen and stored at −80°C until further use. Serum LH concentrations were measured with a rodent LH ELISA kit #ERK R7010 from Endocrine Technologies Inc. (Newark, CA, USA) according to manufacturers' instructions.

### Laser capture microdissection

Brain fixation, preparation of sections for the subsequent laser capture microdissection (LCM) and microarray profiling were performed as reported elsewhere (Khodosevich et al., 2007; Vastagh et al., 2015). Briefly, metestrous (*n* = 6) and proestrous female (*n* = 6) mice were deeply anesthetized and perfused transcardially with 80 ml 0.5% paraformaldehyde followed by 20% sucrose. For microdissection, 7 μm thick coronal brain sections were cut. Sections were mounted on PEN-membrane slides (Zeiss, Jena, Germany), processed further for laser microdissection. Uniform and representative sampling of the entire GnRH neuronal population was performed using LCM performed on a PALM Microbeam system (Carl Zeiss Microimaging Gmbh, Jena, Germany) which was equipped with an epifluorescent setup. About 250 GFP-positive neurons were dissected per animal from 80 to 100 consecutive sections to generate GnRH cell samples from each brain.

### RNA isolation

GnRH cell samples (metestrous: *n* = 6, proestrous: *n* = 6) collected with LCM were incubated in 200 ml lysis buffer at 56°C for 3 h. RNA was isolated from the lysate by proteinase K/acid phenol method (Khodosevich et al., 2007), then precipitated by adding isopropanol (Sigma-Aldrich) and 20 μg glycogen (Thermo Fischer Scientific, Waltham, MA, USA) at −20°C for 30 min followed by centrifugation at 14,000 g at 4°C. The pellet was washed in 70% ethanol, air-dried and resuspended in water. The remaining genomic DNA was eliminated by treatment the mixture with 1U of RNase-free DNase-I (Thermo Fischer Scientific). RNA was purified using RNeasy MinElute Cleanup kit (Qiagen, Hilden, Germany). Total RNA was eluted with 14 μl of ribonuclease-free water. For the analysis of RNA integrity, RNA was isolated from the tissue of the medial preoptic area, and measured using RNA Pico Chip on the 2100 Bioanalyzer (Agilent, Santa Clara, CA, US; Supplementary Figure 2A).

### Whole transcriptome amplification (WTA)

Library preparation and amplification were performed according to the manufacturer's instructions for the WTA2 kit (Sigma-Aldrich). In the first step of the WTA2 protocol, the RNA was reverse transcribed implementing non self-complementary and quasi-random 3′ and universal 5′ primers. The newly synthetized single strands were the templates for annealing and extension steps. When the SYBR Green signal reached a plateau, the reaction was stopped. The resultant library contained cDNA fragments with size distribution between 100 and 1000 base pairs (data obtained from Agilent Bioanalyzer using DNA 1000 microfluidic chip; Supplementary Figure 2B). The amplified double-stranded cDNA was purified and quantified on a Nanodrop ND-1000 spectrophotometer (Thermo Fisher Scientific).

### Mouse genome 430 PM arrays

Eight microgram of cDNA was fragmented by DNase I and biotinylated by terminal transferase obtained from the GeneChip Mapping 250K Nsp Assay Kit (Affymetrix Inc., Santa Clara, CA, USA). Hybridization, washing, staining, and scanning of Affymetrix Mouse Genome 430 PM Strip arrays were performed following the manufacturer's recommendations. The Mouse Genome 430 PM Strip array allows the analysis of 34,325 well-annotated genes using 45,123 distinct probe sets. Scanned images (DAT files) were transformed into intensities (CEL files) using the AGCC software (Affymetrix). RMA analysis was performed by means of the statistical analysis software Partek Genomics Suite (Partek Inc., St. Louis, MO, USA) to obtain probe set level expression estimates.

### Bioinformatics and pathway analysis

All statistical and data mining works were performed in R-environment (R Core Team, 2016) with Bioconductor packages (Huber et al., 2015). Quality assessment of microarrays (*n* = 12) was performed using affyQCReport (Kauffmann et al., 2009). For quality control data regarding RNA integrity, see Supplementary Figure 3. Raw microarray data were pre-processed for analysis using RMA (Robust Multi-Array Average (Irizarry et al., 2003). Fold change (FC) estimation and difference analysis of gene expression were based on linear models combined with Bayesian methods using limma package (Ritchie et al., 2015). FC was calculated from normalized and log_2_ transformed gene expression microarray data for each probe sets. The obtained *p*-values were adjusted by the FDR-based method. The following cut-off criteria were applied on the differentially expressed genes (DEG): |FC| >1.6; and adjusted *p*-value (*p*_adj_) < 0.05. The top 10 differentially expressed genes (up- and down-regulated in proestrus, respectively) are shown in Supplementary Table 1.

For over-representation analysis, the list of DEGs was analyzed using the web-based functional annotation tool DAVID Bioinformatics Resources 6.7 (https://david.ncifcrf.gov) at default settings (Huang da et al., 2009a,b).

For visualization of biological pathways, the data matrix of log_2_ transformed and RMA-normalized microarray data were analyzed using a public web server Graphite Web (Sales et al., 2013). Of the available analytical methods, Signaling Pathway Impact Analysis (SPIA) was used. Over the classical probabilistic components, the SPIA also applies statistical systems biology approaches: it takes into consideration (1) the extent of the expression changes of each gene (2) the position of the DEG within a pathway, (3) the topology of the pathway, (4) the type of interaction between the genes to identify significantly impacted pathways where the total net accumulated perturbation in the pathway (tA) can be calculated (Tarca et al., 2009) The level of significance was 0.05 using FDR correction. Interacting genes of the significant neurotransmitter signaling pathways (pGFDR < 0.05) were visualized using Cytoscape (ver. 3.2.1) open source software platform.

### Quantitative real-time PCR studies

For quantitative real-time PCR (qPCR) investigations of LCM-derived GnRH samples (proestrous females *n* = 6, metestrous females *n* = 5) RNA isolation and WTA were performed as described in the previous section. Amplified and column-purified cDNA was used as template for qPCR. Whole transcriptome-amplified cDNA from LCM samples were diluted in 0.1x TE buffer for qPCR investigation. Inventoried TaqMan assays were used to confirm microarray results by qPCR. Each assay consisted of a FAM dye-labeled TaqMan MGB probe and two PCR primers. Thermal cycling conditions of the qPCR were as follows: 2 min at 50°C and 10 min at 95°C, followed by 40 cycles of 15 s at 95°C and 1 min at 60° C using ViiA 7 real-time PCR platform (Thermo Fisher Scientific). Differential expression of genes was calculated by the 2^−ΔΔCt^ method (Livak and Schmittgen, 2001) using GAPDH gene as a reference. Student's *t*-test were used as a statistical method in comparison of gene expression of the two groups (proestrus: *n* = 6; metestrus: *n* = 5).

## Results

In this study, we analyzed characteristic changes in neurotransmitter signaling of GnRH neurons—regulation of receptor subunits and downstream elements of the signaling cascades at the level of gene expression—by comparison of the neurotransmitter signaling-related elements of the whole transcriptome of GnRH neurons obtained from intact, metestrous and proestrous GnRH-GFP transgenic mice, respectively.

### Over-representation analysis

The functional annotation tool DAVID Bioinformatics Resources was used to discover the most relevant gene ontology (GO) annotations. GO categories were differentially represented (FDR adjusted *p* < 0.05) as follows: GO:0005874~*microtubule*, GO:0000166~*nucleotide binding*; GO:0016071~*mRNA metabolic process*; GO:0031988~*membrane-bounded vesicle*; GO:0007268~*synaptic transmission*; GO:0044456~*synapse part*.

### Differential expression of genes involved in neurotransmitter signaling

**Figure 1:**
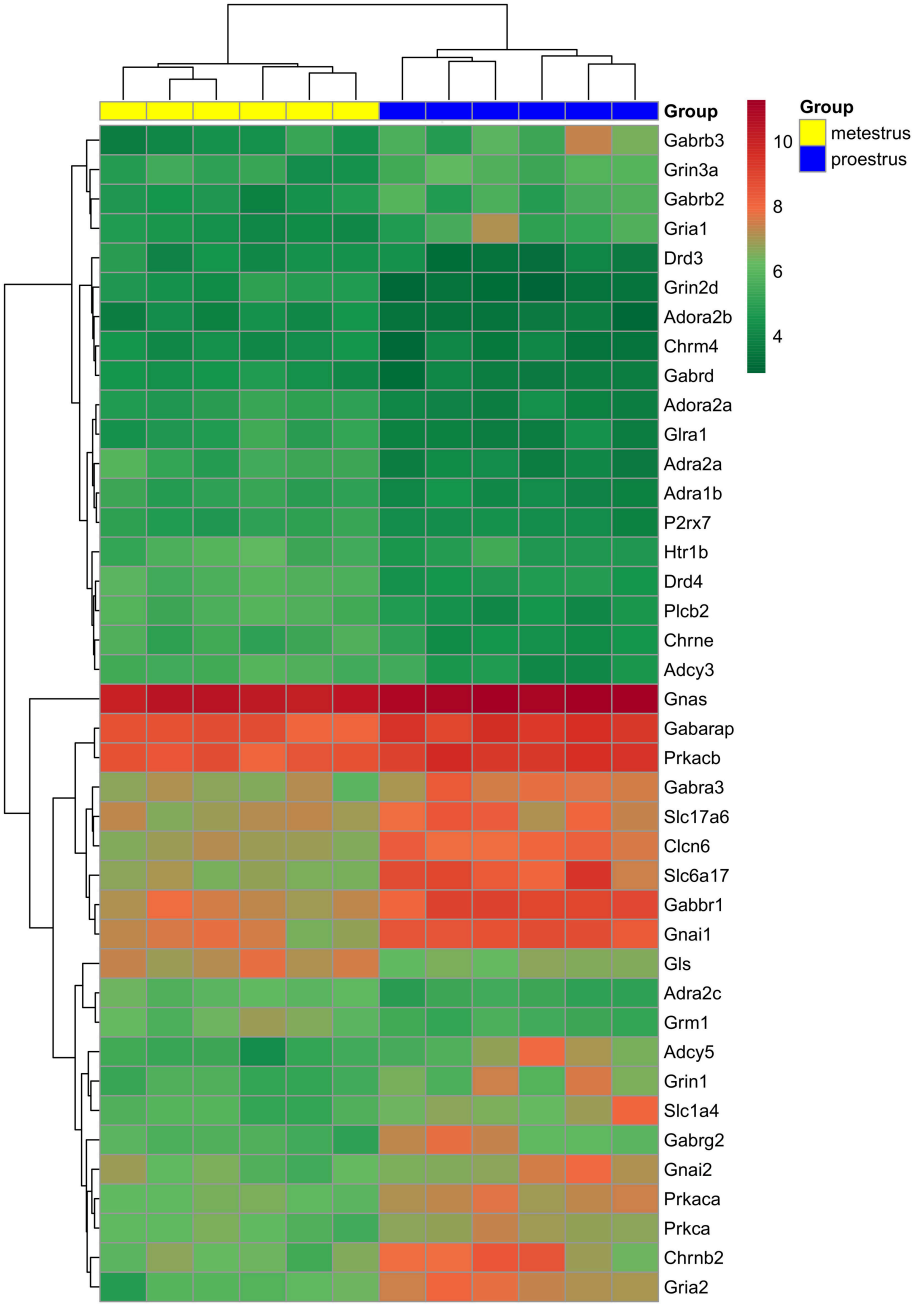
**Hierarchical clustering of genes and microarray experiments**. Expression levels of the genes involved neurotransmitter signaling (see also Table 1) are visualized on a heat map. The rows represent differentially expressed probe sets with corresponding gene symbols on the right. The expression level of each probe is color coded: for decoding, see the color key. The individual samples are shown as columns. The six proestrous and metestrous samples are coded in blue and yellow, respectively.

Analysis of microarray data revealed differentially expressed genes associated with various neurotransmitter signaling mechanisms. Receptor subunits of GABAergic, glutamatergic, and cholinergic communication, as well as receptors participating in adrenergic, serotonergic, dopaminergic, glycinergic, adenosinergic, and purinergic neurotransmission exhibited significant changes (FC > 1.6; adjusted *p* < 0.05) in gene expression levels. In addition, genes encoding G-proteins and downstream effectors as well as neurotransmitter/amino acid transporters of the solute carrier family also showed differential expression (Figure 1 and Table 1).

**Table 1:** **Differentially expressed genes involved in neurotransmitter signaling of GnRH neurons**.

**Upregulated genes in proestrus**					
**Affymetrix ID**	**AE**	**Symbol**	**Description**	**FC**	**adj** ***p-*****value (FDR)**
**CHOLINERGIC SIGNALING**					
1420744_PM_at	123.0	Chrnb2	cholinergic receptor, nicotinic, beta polypeptide 2 (neuronal)	2.65	2.17E-02
**GABAergic SIGNALING**					
1455021_PM_at	269.3	Gabbr1	gamma-aminobutyric acid (GABA) B receptor, 1	2.80	5.95E-04
1421263_PM_at	145.2	Gabra3	gamma-aminobutyric acid (GABA) A receptor, subunit alpha 3	1.90	1.77E-02
1428205_PM_x_at	30.9	Gabrb2	gamma-aminobutyric acid (GABA) A receptor, subunit beta 2	1.97	1.36E-02
1435021_PM_at	35.3	Gabrb3	gamma-aminobutyric acid (GABA) A receptor, subunit beta 3	3.05	1.53E-02
1418177_PM_at	76.6	Gabrg2	gamma-aminobutyric acid (GABA) A receptor, subunit gamma 2	2.20	2.96E-02
1416937_PM_at	481.4	Gabarap	gamma-aminobutyric acid receptor associated protein	1.82	4.36E-03
**G-PROTEINS AND DOWNSTREAM EFFECTORS**					
1427510_PM_at	241.6	Gnai1	guanine nucleotide binding protein (G protein), alpha inhibiting 1	2.59	1.49E-03
1435652_PM_a_at	98.2	Gnai2	guanine nucleotide binding protein (G protein), alpha inhibiting 2	1.88	4.71E-02
1450186_PM_s_at	1687.4	Gnas	GNAS (guanine nucleotide binding protein, alpha stimulating) complex locus	1.73	8.27E-04
1455296_PM_at	58.0	Adcy5	adenylate cyclase 5	2.70	1.84E-02
1450519_PM_a_at	109.1	Prkaca	protein kinase, cAMP dependent, catalytic, alpha	2.06	5.66E-04
1420611_PM_at	496.7	Prkacb	protein kinase, cAMP dependent, catalytic, beta	1.86	1.22E-03
1427562_PM_a_at	87.5	Prkca	protein kinase C, alpha	1.78	7.51E-03
**GLUTAMATERGIC SIGNALING**					
1421970_PM_a_at	99.7	Gria2	glutamate receptor, ionotropic, AMPA2 (alpha 2)	3.13	1.35E-03
1435239_PM_at	30.6	Gria1	glutamate receptor, ionotropic, AMPA1 (alpha 1)	2.36	2.84E-02
1437968_PM_at	65.0	Grin1	glutamate receptor, ionotropic, NMDA1 (zeta 1)	2.24	2.73E-02
1438866_PM_at	39.9	Grin3a	glutamate receptor ionotropic, NMDA3A	1.80	2.11E-02
**SOLUTE CARRIER FAMILY**					
1418610_PM_at	173.0	Slc17a6	solute carrier family 17 (sodium-dependent inorganic phosphate cotransporter), member 6	1.77	1.46E-02
1423549_PM_at	74.0	Slc1a4	solute carrier family 1 (glutamate/neutral amino acid transporter), member 4	2.24	1.46E-02
1436137_PM_at	189.0	Slc6a17	solute carrier family 6 (neurotransmitter transporter), member 17	3.55	1.30E-03
**OTHERS**					
1422314_PM_at	169.2	Clcn6	chloride channel 6	2.27	2.81E-04
**Downregulated genes in proestrus**					
**ADENOSINERGIC SIGNALING**					
1427519_PM_at	21.7	Adora2a	adenosine A2a receptor	0.50	7.47E-04
1450214_PM_at	13.7	Adora2b	adenosine A2b receptor	0.59	7.64E-03
**ADRENERGIC SIGNALING**					
1422183_PM_a_at	24.0	Adra1b	adrenergic receptor, alpha 1b	0.49	5.18E-04
1423022_PM_at	25.0	Adra2a	adrenergic receptor, alpha 2a	0.37	5.07E-04
1422335_PM_at	50.0	Adra2c	adrenergic receptor, alpha 2c	0.56	1.22E-03
**CHOLINERGIC SIGNALING**					
1450575_PM_at	15.0	Chrm4	cholinergic receptor, muscarinic 4	0.58	1.20E-02
1420560_PM_at	30.3	Chrne	cholinergic receptor, nicotinic, epsilon polypeptide	0.52	4.98E-03
**DOPAMINERGIC SIGNALING**					
1422278_PM_at	16.0	Drd3	dopamine receptor D3	0.60	4.99E-02
1422829_PM_at	36.7	Drd4	dopamine receptor D4	0.45	8.28E-05
**GABAergic SIGNALING**					
1457763_PM_at	16.3	Gabrd	gamma-aminobutyric acid (GABA) A receptor, subunit delta	0.58	4.98E-03
**G-PROTEINS AND EFFECTORS**					
1421959_PM_s_at	34.1	Adcy3	adenylate cyclase 3	0.50	9.04E-03
1452481_PM_at	32.8	Plcb2	phospholipase C, beta 2	0.42	3.99E-04
**GLUTAMATERGIC SIGNALING**					
1438827_PM_at	118.3	Gls	Glutaminase	0.56	4.53E-03
1425700_PM_at	57.6	Grm1	glutamate receptor, metabotropic 1	0.53	5.78E-03
1421393_PM_at	15.1	Grin2d	glutamate receptor, ionotropic, NMDA2D (epsilon 4)	0.37	1.49E-04
**SEROTONERGIC SIGNALING**					
1422288_PM_at	37.3	Htr1b	5-hydroxytryptamine (serotonin) receptor 1B	0.58	1.14E-02
**PURINERGIC SIGNALING**					
1422218_PM_at	24.0	P2rx7	purinergic receptor P2X, ligand-gated ion channel, 7	0.59	2.20E-03
**OTHERS**					
1422277_PM_at	20.6	Glra1	glycine receptor, alpha 1 subunit	0.49	2.76E-03

*The list of differentially expressed genes (DEGs) with false discovery rate (FDR) value < 0.05 was screened for genes that are involved in neurotransmitter signaling, i.e., receptor subunits, enzymes, G-proteins. DEGs of the major neurotransmitter/neuromodulatory systems are listed here: GABAergic, glutamatergic, dopaminergic, adrenergic, serotonergic, cholinergic, purinergic, and adenosinergic systems. FC values indicate the changes of expression in the proestrous vs. metestrous GnRH neurons. AE, average expression values at probeset level*.

Significant downregulation was found in gene expression of adenosinergic (*Adora2a, Adora2b*), adrenergic (*Adra1b, Adra2a, Adra2c*) serotonergic (*Htr1b*), purinergic (*P2rx7*), and cholinergic (*Chrm4, Chrne*) receptors or their subunits, respectively, by microarray data analysis. Similarly, the dopaminergic receptor *Drd3* and *Drd4* exhibited lower mRNA level in proestrus. The *Gabbr1* coding for GABA-B receptor 1 and the GABA-A receptor subunit *Gabra3, Gabrb2, Gabrb3, Gabrd, Gabrg2* were upregulated. The gene of the GABA-A receptor associated protein, *Gabarap* were also upregulated. Other GABA-A receptor subunit, the *Gabrd* showed downregulated gene expression. Receptor subunits of the glutamatergic signaling were upregulated (*Grin3a, Gria1, Gria2*, and *Grin1*) whereas *Grin2d*, the metabotropic glutamate receptor *Grm1* and glutaminase (*Gls*) were downregulated. The expression level of the nicotinic cholinergic receptor beta polypeptide 2 (Chrnb2), as well as several heterotrimeric G-protein alpha subunits (*Gnai1, Gnai2, and Gnas*) were increased. Genes of downstream primary effector proteins were expressed at either higher (*Adcy5*) or lower level (*Adcy3, Plcb2*) in proestrus. The secondary effector *Prkaca, Prkacb*, and *Prkca* were upregulated. As far as the solute carrier families are concerned, the glutamate *Slc1a4*, the vesicular glutamate transporter *Slc17a6* and sodium-dependent vesicular transporter *Slc6a17* exhibited higher expression level in GnRH neurons in proestrus. Expression of *Clcn6* and *Glra1* genes, both involved in cellular chloride ion transport, were up- or down-regulated, respectively.

### Validation of the microarray data

**Figure 2:**
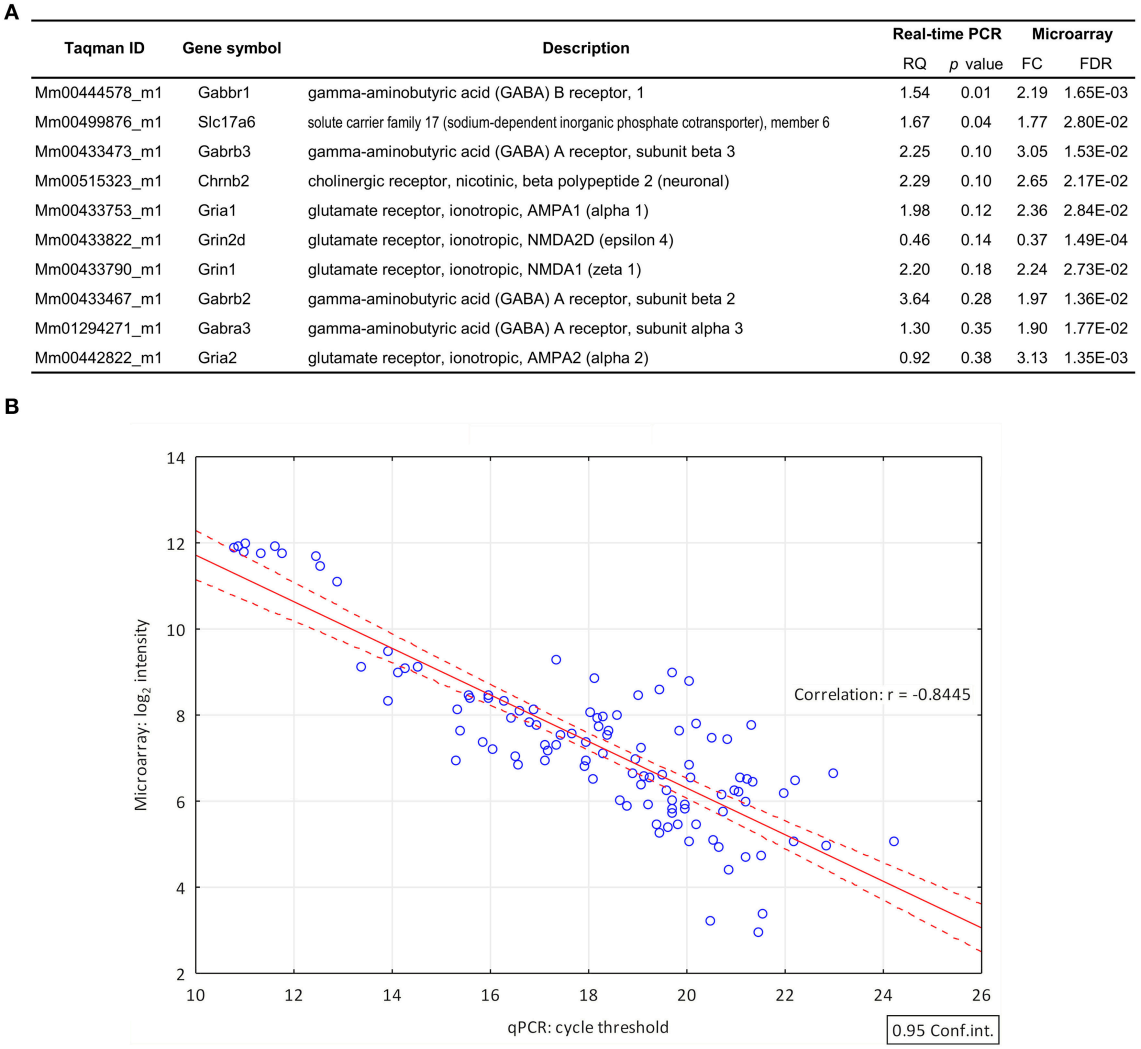
**Validation of differential gene expression data by quantitative real-time PCR. (A)** List of genes (FC > 1.6; FDR < 0.05) selected for validation of their differential expression by qPCR. The qPCR confirmed the differential expression of 8 genes. RQ, relative quantity **(B)** The correlation coefficient (Pearson's *r* = −0.8445) indicates the quantitative correlation between the log_2_ transformed and normalized spot intensity values of microarray hybridizations and the cycle threshold (Ct) value of the qPCR investigations. X-axis, cycle threshold value; Y-axis, normalized microarray expression values.

TaqMan real-time PCR was used for validation of microarray data (Figure 2A). Out of the 9 selected target genes, representing GABA, glutamate and acetylcholine neurotransmission, the differential expression of 8 genes was confirmed (*Slc17a6, Grin1, Gria1, Chrnb2, Gabrb2, Gabbr1, Gabrb3, Gabra3*) and 6 of them exhibited FC >1.5 (*Slc17a6, Grin1, Gria1, Chrnb2, Gabrb2, and Gabbr1*). We found significant differences in gene expression of GABAB1 and Slc17a6 genes by qPCR. Differences between other targets were not significant due to the high standard deviation of individual samples. The correlation coefficient (Pearson's *r* = −0.8445) indicates the quantitative interrelation between microarray and real-time PCR data (Figure 2B).

### Pathway analyses

High throughput analysis of microarray data from 6 proestrous and 6 metestrous females by Signaling Pathway Impact Analysis (False Discovery Rate, FDR of DEGs < 0.05) identified 32 KEGG and 34 REACTOME pathways (adjusted pG using FDR, pGFdr < 0.05). Three of the KEGG pathways (*GABAergic, cholinergic, and dopaminergic signaling*) and two of the REACTOME pathways (*Neurotransmitter receptor binding and downstream transmission in the postsynaptic cell; Activation of NMDA receptor upon glutamate binding and postsynaptic events*) were strongly associated with neurotransmitter signaling and/or its postsynaptic events (Table 2).

**Table 2:** **Significant pathways involved in neurotransmitter signaling identified by Signaling Pathway Impact Analysis (SPIA)**.

**Database**	**Pathway**	**pSize**	**NDE**	**pNDE**	**tA**	**pPERT**	**pG**	**pGFdr**	**pGFWER**	**Status**
REACTOME	Neurotransmitter Receptor Binding And Downstream Transmission In The Postsynaptic Cell	109	41	9.11E-05	250.53	5.00E-06	1.03E-08	3.37E-06	3.37E-06	Activated
REACTOME	Activation of NMDA receptor upon glutamate binding and postsynaptic events	26	10	3.72E-02	50.73	5.00E-06	3.07E-06	5.05E-04	1.01E-03	Activated
KEGG	GABAergic synapse	64	25	1.08E-03	−4.55	2.20E-02	2.76E-04	5.10E-03	3.26E-02	Inhibited
KEGG	Dopaminergic synapse	116	40	8.73E-04	−0.18	9.48E-01	6.70E-03	3.55E-02	7.90E-01	Inhibited
KEGG	Cholinergic synapse	91	29	1.40E-02	11.92	7.70E-02	8.45E-03	3.80E-02	9.97E-01	Activated

*Out of significant pathways (pGFdr < 0.05) the neurotransmission signaling-related pathways are shown in the table. Legend to figure: pSize, the number of genes in the pathway; NDE, number of differentially expressed genes in the pathway; pNDE, hypergeometric probability of observing NDE differentially expressed genes in the pathway by chance; tA, observed value of the perturbation score; pPERT, bootstrap probability associated to tA; pG, combined probability of pNDE and pPERT; pGFDR, adjusted pG using False Discovery Rate correction; pGFWER, adjusted pG using Family Wise Error Rate (Bonferroni); STATUS, Inhibition/Activation according to the negative/positive sign of tA*.

#### GABAergic signaling

**Figure 3:**
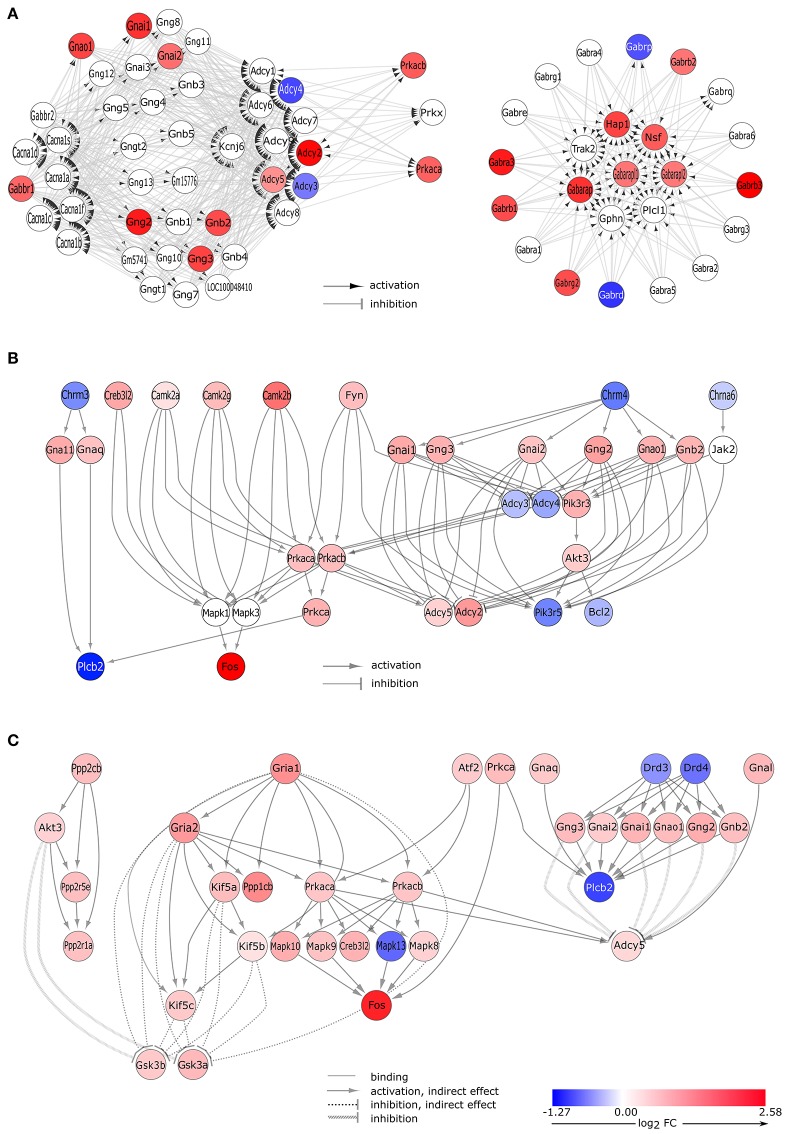
**Gene expression changes in neurotransmitter signaling pathways. (A)** GABA-ergic synapse pathway. Signaling Pathway Impact Analysis (SPIA) of the microarray data revealed significant enrichment of differentially expressed genes (DEGs) in the “GABAergic synapse” pathway (Bonferroni *p* = 0.005). Nodes and edges of the graph represent genes and their relations, respectively. Colored nodes indicate DEGs with color proportional to log2 fold change values. White node are genes that are not expressed differentially. **(B)** DEGs of the cholinergic synapse pathway. Gene expression data analysis by SPIA revealed differentially expressed genes that are involved in the “cholinergic signaling” pathway (Bonferroni *p* = 0.038). Interaction of differentially expressed genes are presented here. The complete pathway is shown in Supplementary Figure 4. **(C)** DEGs of the dopaminergic synapse pathway. Microarray data analysis by SPIA showed that a significant number of the differentially expressed genes was enriched in the dopaminergic signaling pathway (Bonferroni *p* = 0.035). Interaction of differentially expressed genes are presented here; the complete pathway is depicted in Supplementary Figure 5.

The “GABAergic synapse” KEGG pathway (pGFdr = 5.10E-03) was stated as inhibited (observed value of the perturbation score, tA = −4.555) and involved 25 differentially expressed genes (Table 2). In proestrus, subunits of the GABA-A receptor showed altered gene expression level (up-regulated: *Gabra3, Gabrb1, Gabrb2, Gabrb3, Gabrg2*; down-regulated: *Gabrd*). The GABA-B receptor 1 (*Gabbr1*) and several downstream G-proteins (*Gnai1, Gnai2, Gnb2, Gng2, Gng3, Gnao1*), and cAMP-dependent protein kinase C (*Prkaca, Prkacb*) were up-regulated. The adenylate cyclases involved in the GABA-B signaling were either upregulated (*Adcy2, Adcy5*) or downregulated (*Adcy3, Adcy4*) in proestrus (Figure 3A and Supplementary Table 2).

#### Cholinergic signaling

Changes in gene expression levels of the “cholinergic synapse” (pGFdr = 3.80E-02) indicate an activation (tA = 11.92, Table 2) of this KEGG pathway that involved 29 differentially expressed genes (Figure 2B). Nicotinic (*Chrne*) and muscarinic (*Chrm4*) cholinergic receptors were down-regulated in GnRH neurons, with the exception of the nicotinic acetylcholine receptor subunit *Chrnb2* (Table 1) which was up-regulated at proestrus. Upregulation of genes in the signaling cascade (*Gnai1, Gnai2, Gnb2, Gng2, Gng3, Gnao1, Prkaca*, and *Prkacb*) was observed. The Gna11 and Gnaq subunits that form the Gq alpha complex and activate the phospholipase C beta 2 (*Plcb2)*, were up-regulated, whereas *Plcb2* was down-regulated in proestrus. The expression level of the calcium/calmodulin-dependent protein kinase II alpha, beta and gamma (*Camk2a, Camk2g, and Camk2b*, respectively) and *Fos* were significantly higher in proestrus (Figure 3B, Supplementary Figure 4 and Supplementary Table 2).

#### Dopaminergic signaling

Dopaminergic signaling pathway (pGFdr = 3.55E-02) in GnRH neurons was stated as inhibited (tA = −0.18) in proestrus revealed by SPIA (Table 2, Figure 3C, and Supplementary Figures 4, 5). According to the microarray data, the expression level of the dopaminergic receptor D3 and D4 (*Drd3* and *Drd4*) was significantly decreased as compared to metestrus. Up-regulation of G-proteins (*Gnai1, Gnai2, Gnb2, Gng2, Gng3, and Gnao1*) and protein kinases (*Prkaca* and *Prkacb*) have been already shown at the GABAergic and cholinergic signaling cascades. Adenylate cyclase (*Adcy5*), inhibited by the upstream G-protein complex, was up-regulated, whereas *Plbc2* activated at protein level, was down-regulated. The glutamatergic pathway interferes with the dopaminergic signals: the upregulation of AMPA 1 and 2 (*Gria1, Gria2*) receptors are linked with the upregulation of the Akt/Gsk3 pathway: kinesin family members *Kif5c, Kif5b*, and *Kif5* indirectly, whereas *Akt3*, from the dopaminergic side, directly inhibits *Gsk3a* and *Gsk3b*.

#### Neurotransmitter receptor binding and postsynaptic signaling

**Figure 4:**
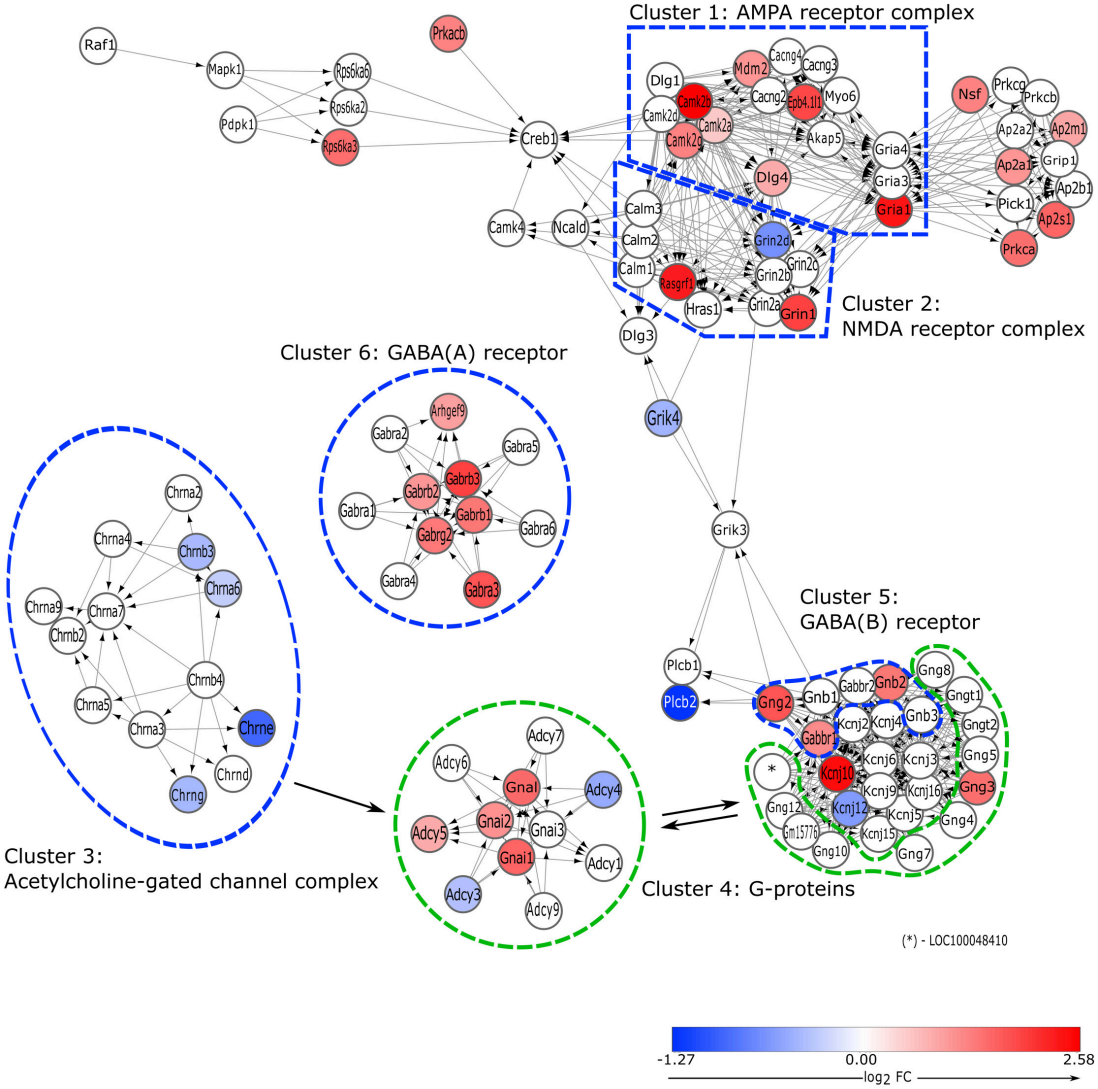
**Overview of gene expression changes in the elements of postsynaptic signaling cascades**. Pathway analysis revealed significant activation of a REACTOME pathway called' Neurotransmitter Receptor Binding and Downstream Transmission in the Postsynaptic Cell' (Bonferroni, *p* = 3.37E-06). Genes that are involved in the various neurotransmitter systems are hierarchically clustered according to their strength of interconnectivity and perturbance level. Clusters were identified as follows: 1. AMPA receptor complex; 2. NMDA receptor complex; 3. Acetylcholine-gated receptor complex; 4. G-proteins; 5. GABA **(B)** receptor; 6. GABA **(A)** receptor.

An activated pathway (tA = 250.53) called “*Neurotransmitter Receptor Binding and Downstream Transmission in the Postsynaptic Cell*” (pGFdr = 3.37E-06) was identified in the proestrous GnRH neurons (Table 2 and Figure 4). The majority of the pathway is built up by hierarchically numbered clusters—highly interconnected regions—as defined by the MCODE Cytoscape plugin. Within the top relevant cluster #1 significant enrichment of genes in the “ionotropic glutamate receptor complex” (*Gria1, Dlg4*; GO; FDR = 5.37E-16) and “calmodulin binding” (*Camk2a, Camk2b, Camk2g*; GO; FDR = 2.71E-06) terms were found. Genes of the cluster #2 were elements of “NMDA receptor complex” (GO; 6.23E-12), in which *Grin1* and *Rasgrf1* were up-, whereas *Grin2d* was downregulated. In cluster #3, which is specific for “acetylcholine-gate channel complex” (GO, FDR = 1.16E-23), acetylcholine receptor subunits (*Chrne, Chrnb3, Chrna6*) were downregulated. Cluster #4 is functionally connected with several other sub-networks (glutamatergic, cholinergic, and GABAergic receptors). This cluster is built up by heterotrimeric G-proteins that involves increased mRNA levels of alpha subunit *Gnai1, Gnai2, Gnai3, Gnal*, and adenylate cyclases of which *Adcy3* and *Adcy4* are downregulated, *Adcy5* is upregulated. Cluster #5 and #6 are parts of the GABA-B and GABA-A receptor complexes, respectively, where *Gabbr1, Gabrb1, Gabrb2*, and *Gabrb3* genes are upregulated.

## Discussion

Fast synaptic neurotransmission is mediated by postsynaptic ionotropic receptors (Greengard, 2001). GABA and glutamate, the two main neurotransmitter systems, play a fundamental role in the regulation of GnRH neurons (Nikolarakis et al., 1988; Donoso et al., 1992; Ottem et al., 2002; Iremonger et al., 2010; Herbison and Moenter, 2011) and the control of ovulation (Christian and Moenter, 2008).

### Gabaergic signaling

In the previous decades, it was shown that hypothalamic GnRH neurons receive functional GABAergic primary afferent inputs (Leranth et al., 1985; Adler and Crowley, 1986; Herbison and Dyer, 1991; Kimura and Jinnai, 1994; Jung et al., 1998; Sim et al., 2000; DeFazio et al., 2002; Temple and Wray, 2005; Zhang et al., 2009). The estrous cycle is characterized by profound changes in GABA transmission to GnRH neurons (Herbison, 1997): prior the LH surge the mRNA level of the GABA synthetizing enzyme GAD67 is significantly reduced (Herbison et al., 1992) which exhibits E2-dependent temporal pattern in the territory of the AVPV (Curran-Rauhut and Petersen, 2002). The release of GABA also falls significantly prior to and during the time of estrogen-induced LH surges (Jarry et al., 1992; Tin-Tin-Win-Shwe et al., 2004). This drop of GABA level before the surge seems essential for the positive feedback (Herbison and Dyer, 1991; Kimura and Jinnai, 1994).

Expression of the α1-3, α5, β1-3, and γ2 subunits of the GABA_A_ receptor were detected in the GnRH neurons of adult female mice earlier (Sim et al., 2000). We found an increased mRNA expression level of the α1, β1-3, and γ2 subunits and in the proestrous female. Although a GnRH neuron-specific γ2 knock-out mice exhibited normal fertility (Lee et al., 2010), higher level of this subunit can increase the conductance and sensitivity of the GABA_A_ receptors (Brickley et al., 1999). The increased mRNA level of the GABA_B_ receptor 1 and that of several GABA_A_ subunits might also indicate the upregulation of these receptors in response of the dropping GABA level in proestrus. However, the observed negative value of the perturbation score (tA = −4.55) indicates a repressed status of the complete GABAergic synapse pathway in GnRH neurons, probably due to downregulation of genes (effector proteins i.e., *Adcy4, Adcy3, Plcb2*), downstream to the GABA receptors in the signaling pathway. Recent functional data support the view that net GABA effects might be determined by the balance of the excitatory GABA_A_ (Herbison and Moenter, 2011) and inhibitory GABA_B_ (Zhang et al., 2009; Liu et al., 2011) receptor-mediated tones on GnRH neurons, even *in vivo* (Constantin et al., 2013). We showed higher expression of both type of the GABA receptors and a repressed status of the GABAergic pathway suggesting an increased sensitivity for the ligand and inhibited signaling cascade that might play a role in the facilitation of the GnRH surge.

### Glutamatergic signaling

GnRH neurons express ionotropic AMPA (α-amino-3-hydroxy-5-methyl-4-isoxazolepropionic acid) and NMDA (N-methyl-D-aspartate) receptors (Gore et al., 1996; Spergel et al., 1999; Ottem et al., 2002) and their antagonists block the LH surge (López et al., 1990, 1992; Brann and Mahesh, 1991; Ping et al., 1997) indicating a prominent role of glutamate in the positive feedback regulation. At the time of the GnRH/LH surge, the number of contacts that express both glutamate and GABA transporters (Ottem et al., 2004), as well as the level of glutamate increases in the neighborhood of the GnRH cell bodies (Ping et al., 1994; Jarry et al., 1995). The expression of AMPA receptors changes at the level of the somata in steroid-induced LH surge model of young female rats, although, very few (1%) GnRH neurons co-synthetize AMPA receptors and Fos in the morning of proestrus. In contrast, the increase in the number of Gria1/Fos positive (25%) and Gria2/Fos positive (71%) GnRH neurons coincides with the increase and peak of LH (Bailey et al., 2006). In concert with these data, we found that expression of the AMPA1 and AMPA2 receptor subunits (*Gria1* and *Gria2*, respectively) increased by the time of LH surge. Though the percentage of NMDAR1-immunopositive GnRH neurons does not change in the mouse (Adjan et al., 2008), we found that the expression level of NMDA subunits *Grin1* and *Grin3a* increased significantly in proestrus. The higher expression level of these receptor subunits and enrichment of genes in the ionotropic glutamate receptor complex suggest an enhanced glutamate receptor signaling in GnRH neurons during the preovulatory GnRH/LH surge.

### Cholinergic signaling

In the past decades, acetylcholine (ACh) has been shown to exert a stimulatory effect on the release of the GnRH peptide *in vitro* (Fiorindo and Martini, 1975; Richardson et al., 1982). Cholinergic afferents to hypothalamic GnRH neurons have been detected at both light and electron microscopic levels in the rat (Turi et al., 2008). Studies using perifused hypothalamic and immortalized GnRH (GT1-7) neurons indicated that selective activation of the nicotinic ACh receptor stimulated, in contrast, muscarinic receptor-specific activation inhibited GnRH release (Krsmanovic et al., 1998). *In vivo* pharmacological experiments in rats suggested an estrous cycle-dependent stimulation of GnRH release by selective muscarinic antagonists (Koren et al., 1992) induced via the M4 receptor subtype encoded by the *Chrm4* gene. We found a significantly downregulated *Chrm4* expression in proestrus and an upregulated level of *Chrnb2*. The altered regulation of the two subunits and the diverse action of the nicotinic and muscarinic-type ACh receptor activation can promote GnRH release and surge in proestrus.

### Dopaminergic signaling

GnRH neurons receive tyrosine-hydroxylase (TH) positive terminals demonstrated both at light microscopic (Jennes et al., 1983) and ultrastructural levels (Leranth et al., 1988; Chen et al., 1989; Horvath et al., 1993). Dual phenotype kisspeptin-TH positive fibers of AVPV origin apposed to the somata of GnRH neurons can be one of the sources of these dopaminergic inputs (Clarkson and Herbison, 2011). Dopamine inhibited the firing and AVPV-evoked GABA/glutamate postsynaptic currents in ~50% of the GnRH neurons *in vitro* mediated by D1 and D2-like receptors in male and female mice (Liu and Herbison, 2013) showing no evidence of an estrous cycle-dependent modulation when diestrous, proestrous, and estrous female mice were compared in the morning period. In the current study, D2-like dopamine receptors encoded by *Drd3* and *Drd4* genes were found to be down-regulated in proestrous GnRH neurons, suggesting that expression of these genes are affected by the estrous cycle. However, further functional studies are needed to clarify if down-regulation in the expression of the D3 and D4 receptor genes can decrease the inhibitory effect of dopamine on the excitability of the GnRH neurons in the late afternoon period of proestrus.

### Adrenergic signaling

Numerous studies have supported the view for a long time that norepinephrine (NE) exerts a stimulatory effect on GnRH release in the presence of E2 in OVX+E replacement models (Gallo and Drouva, 1979; Ferris et al., 1984; Wise, 1984). Later, NE was found to suppress the excitability of the GnRH neurons acting through both alpha 1 and beta adrenergic receptors with reduced rate of responsive GnRH neurons during proestrus, otherwise exerting a similar effect across the estrous cycle (Han and Herbison, 2008). We found that adrenergic receptors *Adra1b, Adra2a*, and *Adra2c* are significantly downregulated in proestrus compared to metestrus. The decreased mRNA expression level of these receptors might indicate an attenuated catecholamine sensitivity of GnRH neurons specifically in the late afternoon period; prior to and during LH surge.

### Serotonergic signaling

Earlier investigations provided evidence that 5-HT neurons project directly to GnRH neurons in rodents (Jennes et al., 1982; Kiss and Halász, 1985; Wada et al., 2006; Campbell and Herbison, 2007) In the mouse, serotonin (5-HT) exerts a biphasic effect on the excitability of GnRH neurons: both inhibitory and excitatory responses have been demonstrated, mediated by activation of 5-HT1A and 5-HT2A receptors, respectively (Bhattarai et al., 2014). The serotonergic input to GnRH neurons showed an estrous cycle-dependent regulation in the adult female mouse: during proestrus, the serotonergic inhibition was reduced, accompanied by the absence of the biphasic responses. The 5-HT1B receptor also mediates inhibitory neurotransmission (Mathur et al., 2011; Huang et al., 2013) in the CNS. Although the function of the 5-HT1B receptor was not investigated in GnRH neurons to date, we found that it was down-regulated in proestrous GnRH neurons. Compared to other stages of the estrous cycle, 5-HT1 receptor expression is decreased up to 40% in the basal forebrain during proestrus (Biegon et al., 1980) when peaking level of E2 significantly down-regulates 5-HT receptors (Biegon and McEwen, 1982). The current study further supports that a suppression of the inhibitory 5-HT tone takes place in GnRH neurons in proestrus.

### Purinergic signaling

#### P2X receptors

P2X receptors are trimeric, ligand-gated cation channels activated upon binding of extracellular ATP. They are highly permeable to Ca^2+^ and expressed in diverse organs including the brain (Soto et al., 1996; Burnstock, 2013). Purinergic signaling plays a fundamental role in the regulation of hypothalamo-pituitary functions (Stojilkovic, 2009). Expression of P2X2 and P2X4 has been confirmed in GnRH neurons in olfactory placode cultures (Terasawa et al., 2005) where application of ATP helps to synchronize [Ca^2+^]_i_ oscillations. Furthermore, P2X5 and P2X6 receptors are also expressed in GnRH neurons of mice as revealed by double-labeling immunofluorescence (Fu et al., 2009). Interestingly, though the presence of P2X7 receptor has not been confirmed in GnRH neurons to date, the mRNA level of *P2xr7* is significantly down-regulated in proestrous GnRH neurons. A possible explanation of this phenomenon may be the E2-dependent regulation of P2X7R expression during the estrous cycle. Data from literature indicate that treatment with E2 decreases the expression of P2X7 receptor both *in vitro* (Cario-Toumaniantz et al., 1998) and *in vivo* (Xu et al., 2016).

#### P1 receptors

The adenosine receptor 2A and 2B receptors are members of the G protein-coupled receptor (GPCR) family. The A2 adenosine receptors are coupled to G_s_ and G_olf_ family of G proteins stimulating adenylate cyclase activity to increase the intracellular cAMP level (Schwindinger et al., 2010). Expression of *Adora2b* has been shown in hypothalamic GnRH neurons (Todman et al., 2005). In this study we found significant down-regulation of the *Adora2a* and *Adora2b* transcripts in proestrous GnRH neurons. The role and significance of the regulation of adenosine receptor 2 are unknown at present, they may be linked to the maintenance of intracellular cAMP levels along the estrous cycle.

### Methodological considerations

#### Sampling by LCM

LCM is among the best approaches available for cell-type-specific microarray gene expression profiling (Lin et al., 2007; Okaty et al., 2011; Demarest et al., 2012). This methodology helps to preserve the integrity of the GnRH transcriptome and ensures the systematic sampling of the hypothalamic GnRH neurons. Our strategy aimed to minimize the extent of contamination from surrounding cells was as follows: (1) brain sections were cut at 7 μm thickness; (2) during microdissection, the cutting laser were directed strictly along the perikaryon of the GnRH-GFP neurons. While it is not possible to eliminate contamination during sample preparation completely, careful implementation of the steps above results in highly enriched mRNAs isolated from GnRH neurons.

#### Linearity of the RNA amplification

In this work we implemented a relatively new approach for gene expression profiling validated and published earlier (Gonzalez-Roca et al., 2010) showing that “pico profiling” allows an accurate measure of transcript levels from populations as low as 10 cells with insignificant number of false positive or negative hits, using a commercially available TransPlex WTA2 kit from Sigma-Aldrich. Importantly, at the reverse transcription step the quasi-random 3′ and universal 5′ primers resulted in DNA fragments flanked by universal end sequences to ensure the linear amplification of the expressed genes without 3′ and 5′ bias.

#### Validation of the data

High throughput gene expression analysis of GnRH neurons across the estrous cycle was performed for the first time in the present study. A previous study has addressed ion channel expression and E2 regulations in GnRH neurons during negative and positive feedback (Bosch et al., 2013). They showed that E2 treatment up-regulates voltage-gated calcium channel (VGCC) Cav1.3, Cav2.2, and Cav2.3 mRNAs in GnRH neurons. In consistence with their results regarding the expressional changes in the positive feedback period, we have also found a significant increase in the expression of the Cav 2.2, the voltage-dependent N-type calcium channel alpha 1B subunit encoded by Cacna1b gene (FC 1.7; *p*_adj_ < 0.01) in proestrus whereas the increase in mRNA expression the Cav1.3 [Cacna1d] and Cav2.3 [Cacna1e] subunits was not significant. This discrepancy may be due to the different experimental paradigm: Bosch et al. used *ex-vivo* sorted GnRH neurons from OVX mice primed with E2 dose followed by a surge-inducing E2 dose, whereas in the current study naturally cycling intact mice were used.

#### Correlation of mRNA and protein abundances

In this study, differential expression of genes was investigated as alterations in the mRNA abundances. In general, changes of the protein levels should not be well correlated with that of the mRNA levels due to many factors that influence the expression of proteins (ribosomal density, protein half-life, etc.) and the experimental error and noise (Maier et al., 2009; Vogel and Marcotte, 2012). Genome-wide correlations between mRNA and protein expression levels are usually weak (de Sousa Abreu et al., 2009). However, the level of mRNAs expressed differentially significantly correlate better with their proteins compared to genes that are not expressed differentially (Koussounadis et al., 2015) further increasing the confidence of studies applying differential mRNA expression measurements.

## Conclusion

We found considerable differences in the expression of genes encoding neurotransmitter receptors and their effectors in downstream signaling cascades by comparison of GnRH neurons obtained from pro- and metestrous mice, respectively. Signaling systems known to facilitate the GnRH surge (glutamatergic, GABA-A receptor-mediated inputs and cholinergic neurotransmission via nicotinic receptors) become activated, while neuronal inputs that exert inhibitory effects on GnRH release (dopaminergic, serotonergic, adrenergic systems) seem to be negatively regulated at the level of transcripts in proestrus. These complex changes in the gene expression of proestrous GnRH neurons may alter diverse intracellular mechanisms that culminate in the preovulatory GnRH surge.

## Author contributions

CV designed and performed the experiments, analyzed the data and wrote the manuscript. AR executed hybridization and scanning of microarrays. NS carried out the bioinformatical analysis of the microarray data. ZL designed and supervised the project, and wrote the manuscript.

## Funding

This work was supported by the Hungarian Scientific Research Fund (OTKA K100722, OTKA 115984).

### Conflict of interest statement

The authors declare that the research was conducted in the absence of any commercial or financial relationships that could be construed as a potential conflict of interest.
