# Supplementary material for: Altered Expression of Genes Encoding Neurotransmitter Receptors in GnRH Neurons of Proestrous Mice
Source: Front Cell Neurosci. 2016 Oct 7;10:230. doi: 10.3389/fncel.2016.00230 (PMC5054603; doi:10.3389/fncel.2016.00230)
Supplement: Supplementary file 1 [file DataSheet1.docx]

Supplementary Material

Altered expression of genes encoding neurotransmitter receptors in GnRH neurons of proestrous mice

Csaba Vastagh^1*^, Annie Rodolosse^2^, Norbert Solymosi^3^ and Zsolt Liposits^1, 4^

*** Correspondence:**

Corresponding Author: [vastagh.csaba@koki.mta.hu](mailto:vastagh.csaba@koki.mta.hu)

## Supplementary Figures


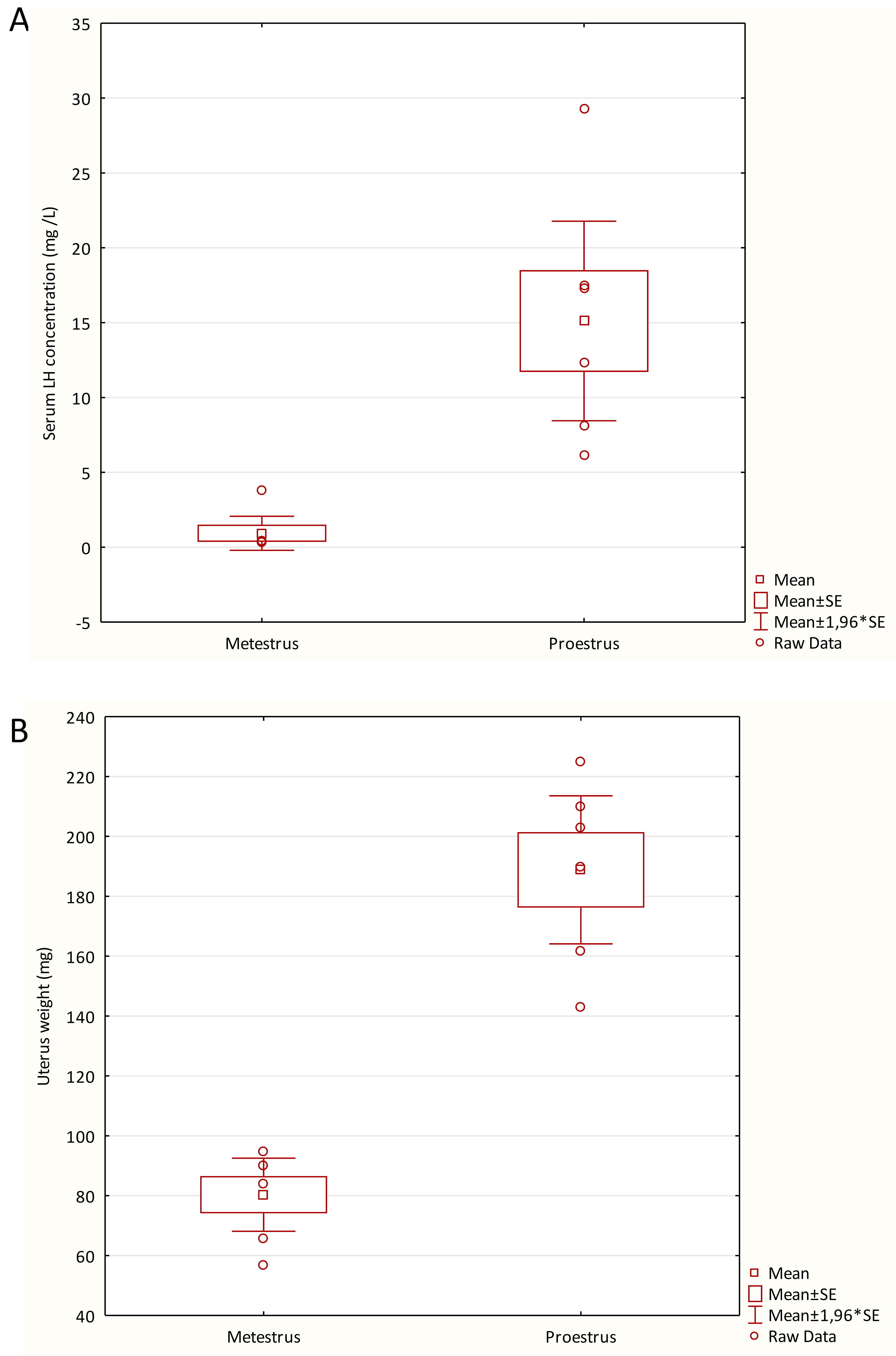


**Supplementary Figure 1**: The graphs illustrate data of serum LH concentrations (**A**) and uterine weights (**B**) measured in metestrous and proestrous female mice.


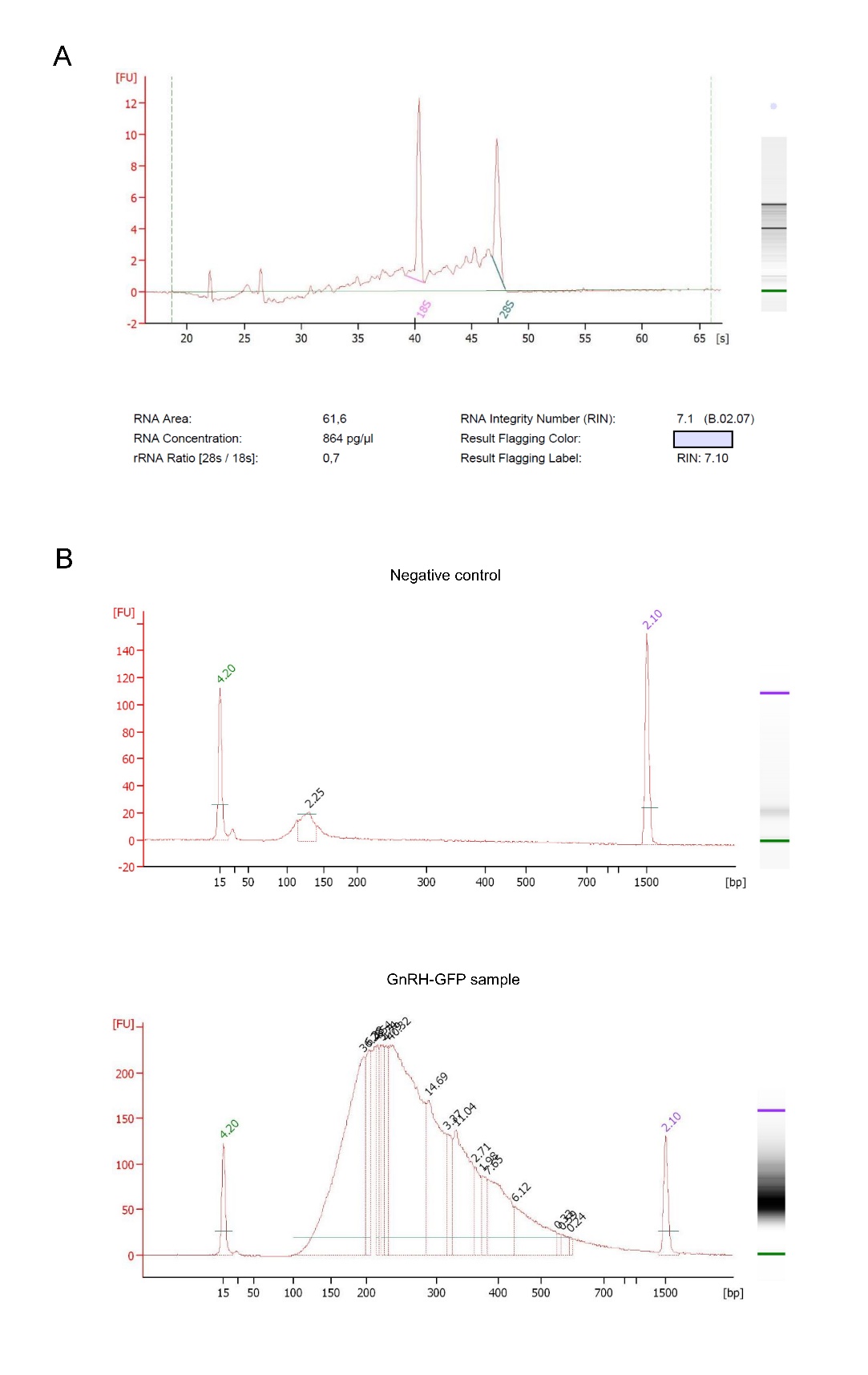


**Supplementary Figure 2.** **(A)** RNA quality control measurement using Bioanalyzer RNA Pico Chip. Total RNA was isolated from the medial preoptic area representing the RNA integrity of LCM-sampled GnRH-GFP neurons. **(B)** Electropherogram of the amplified cDNA library from GnRH-GFP cells after the WTA using Bioanalyzer DNA1000 chip. DNA product size ranges from 100 to 1000 bp. RNA input was omitted from reaction mix of the negative control.

**
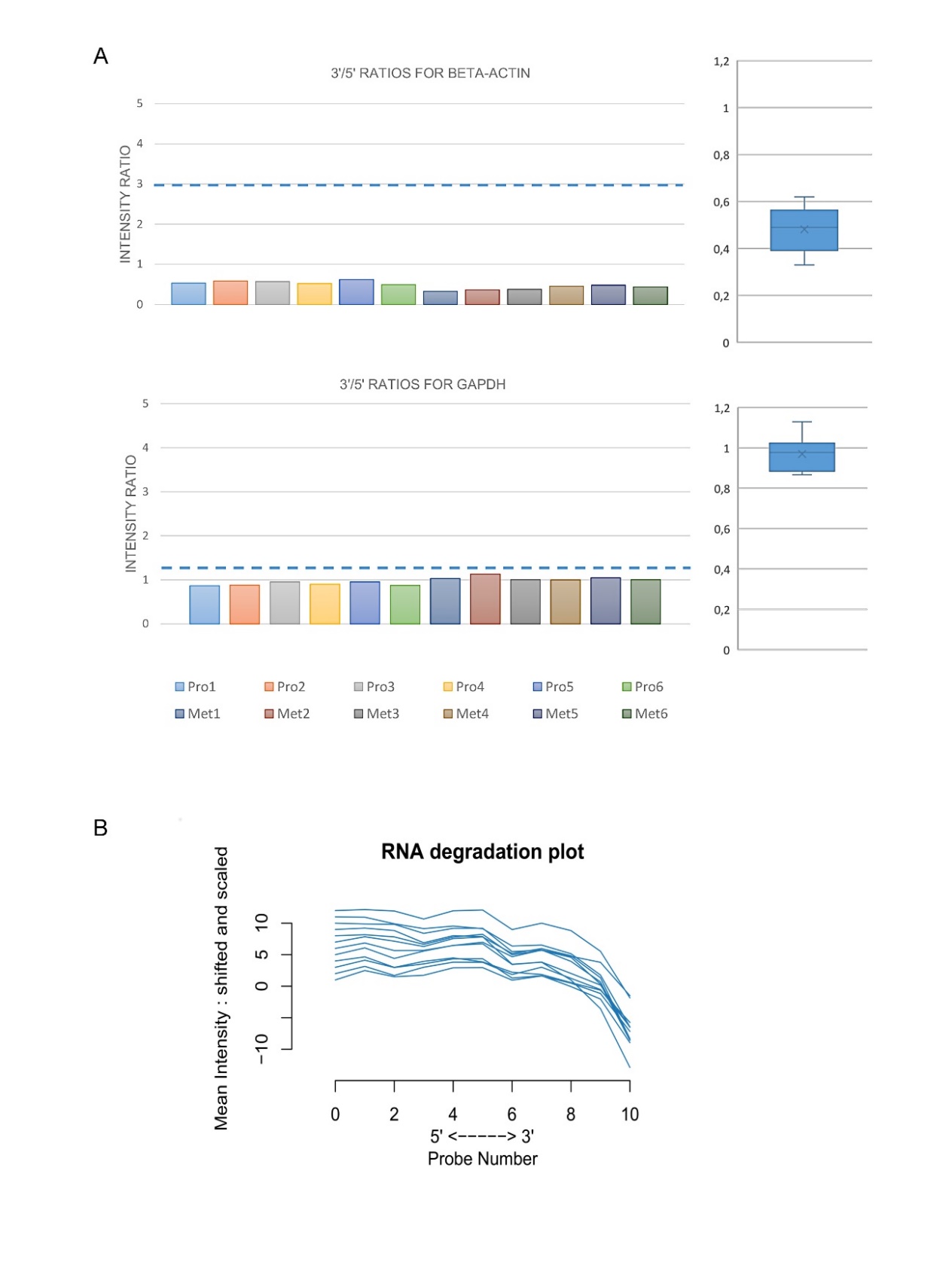
**

**Supplementary Figure 3. (A)** 3'/5' ratios for beta-actin and Gapdh calculated from quality control data of microarray hybridizations. All of the individual data are below the threshold values indicated by scattered lines (values are 3.00 and 1.25 for beta-actin and Gapdh, respectively). **B.** RNA degradation plot values computed from preprocessed data after background correction and quantile normalization. Each array is represented by a single line. All the lines have a similar slope indicating that the RNA used prior to array hybridizations has been handled identically.

**
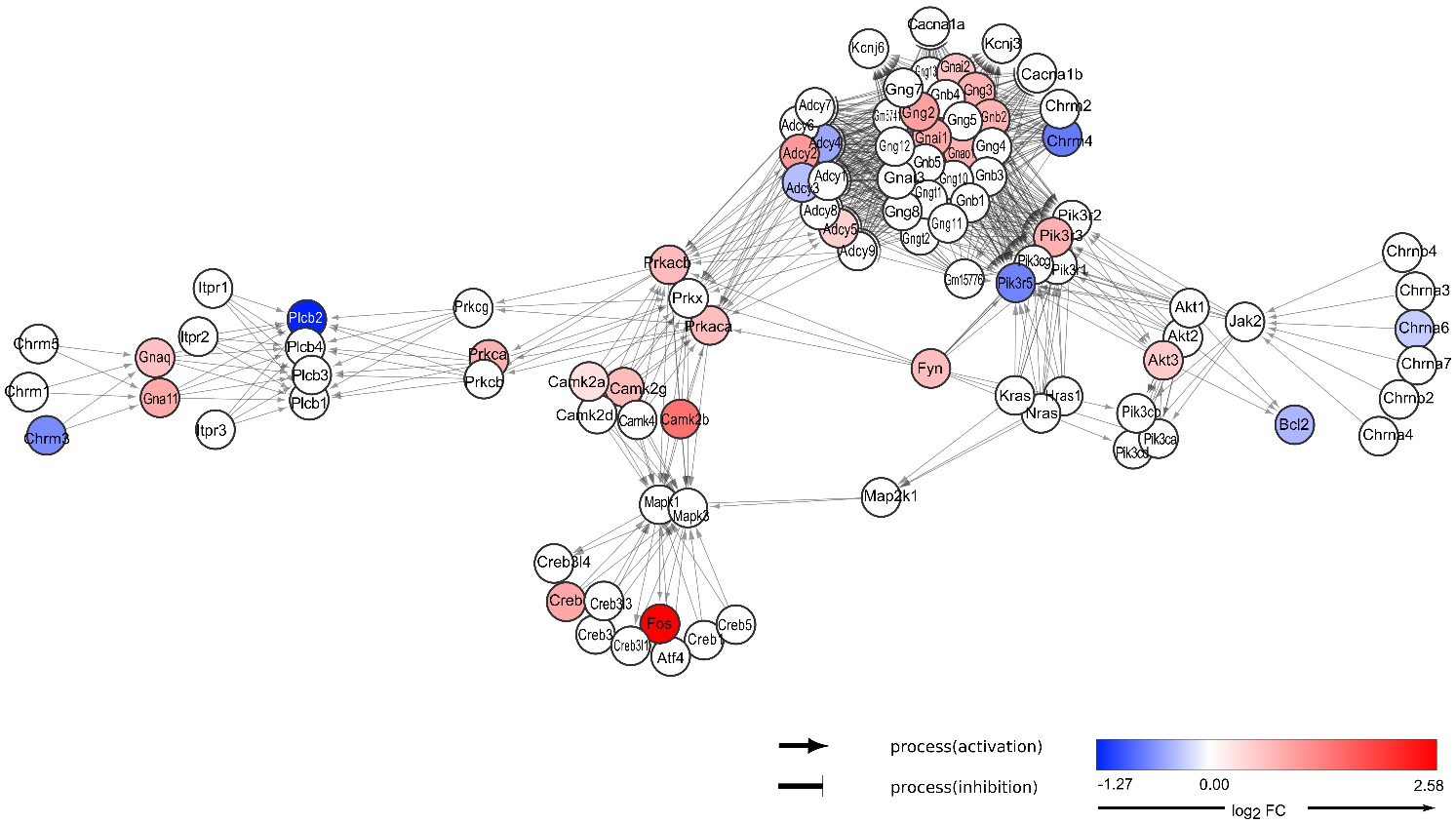
**

**Supplementary Figure 4.** The complete ’cholinergic synapse’ pathway (KEGG) showing the differentially expressed genes


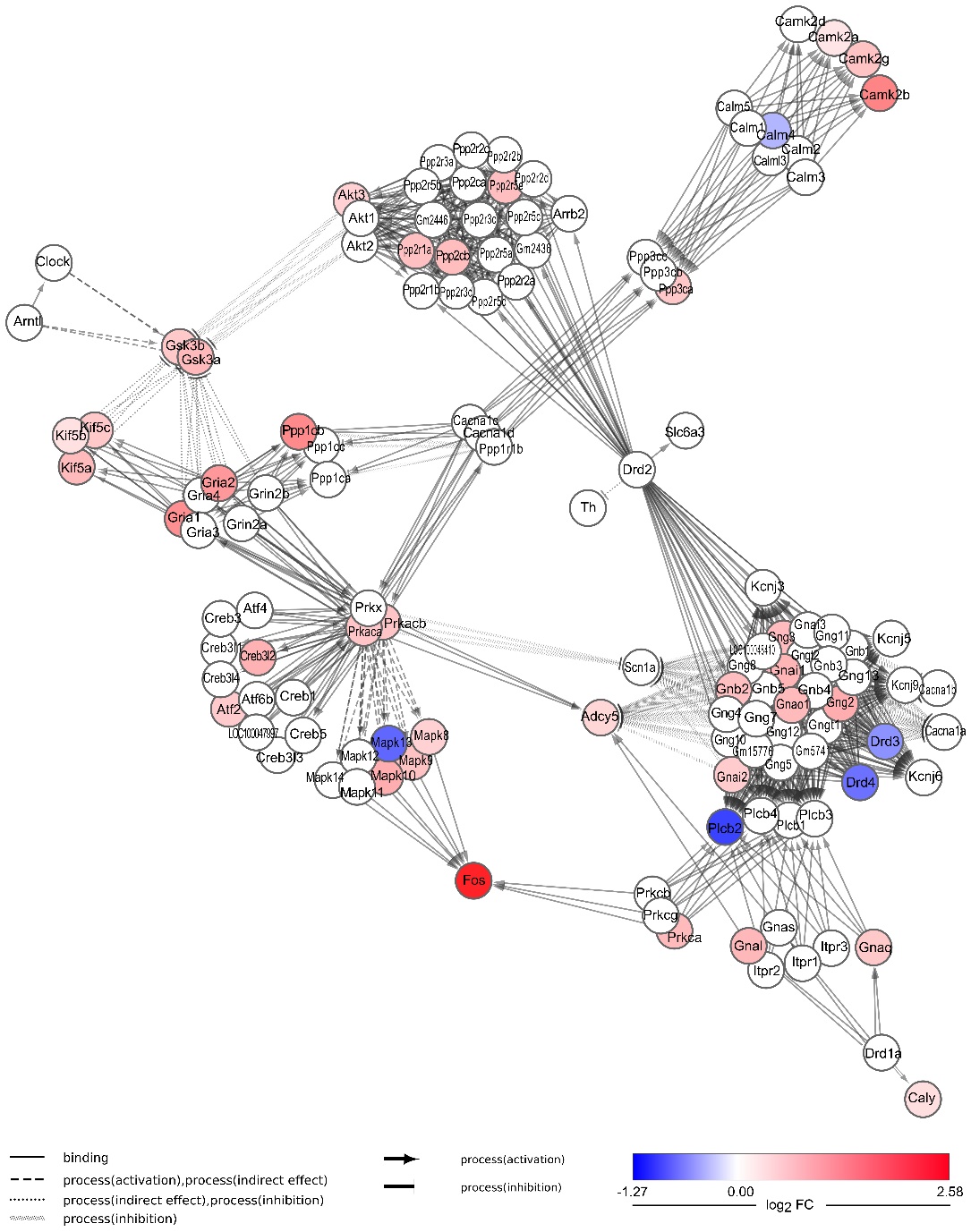


**Supplementary Figure 5.** The complete ’dopaminergic synapse’ pathway (KEGG) showing the differentially expressed genes.

**Supplementary Table 1.** List of the top 10 up and down-regulated genes, indicated by positive and negative log FCs, respectively, in proestrous versus metestrous GnRH neurons.

| **Symbol** | **log FC** | **adj. *p* value** | **Description** |
| --- | --- | --- | --- |
| Cited1 | 3.28 | 4.36E-03 | Cbp/p300-interacting transactivator with Glu/Asp-rich carboxy-terminal domain 1 |
| Gem | 3.18 | 2.70E-02 | GTP binding protein (gene overexpressed in skeletal muscle) |
| Rrad | 2.89 | 2.01E-02 | Ras-related associated with diabetes |
| Fus | 2.84 | 1.68E-03 | fused in sarcoma |
| Srxn1 | 2.80 | 5.65E-03 | sulfiredoxin 1 homolog (S. cerevisiae) |
| Vgf | 2.80 | 4.71E-02 | VGF nerve growth factor inducible |
| 4833410I11Rik | 2.72 | 1.95E-03 | RIKEN cDNA 4833410I11 gene |
| Tfrc | 2.70 | 6.17E-03 | transferrin receptor |
| Arf6 | 2.66 | 1.96E-04 | ADP-ribosylation factor 6 |
| Srxn1 | 2.60 | 4.27E-02 | sulfiredoxin 1 homolog (S. cerevisiae) |
| … | … | … | … |
| Wdr91 | -2.41 | 4.44E-05 | WD repeat domain 91 |
| Defa-rs7 | -2.54 | 1.32E-05 | defensin, alpha, related sequence 7 |
| Ubc | -2.59 | 1.59E-05 | ubiquitin C |
| Tpd52 | -2.60 | 4.55E-05 | tumor protein D52 |
| Pih1d1 | -2.72 | 2.59E-05 | PIH1 domain containing 1 |
| Rassf10 | -2.80 | 8.28E-05 | Ras association (RalGDS/AF-6) domain family (N-terminal) member 10 |
| Pttg1 | -3.04 | 4.15E-07 | pituitary tumor-transforming gene 1 |
| Tubb2c-ps1 | -3.15 | 2.91E-04 | tubulin, beta 2c, psuedogene 1; tubulin, beta 2C; tubulin, beta 2c, pseudogene 2 |
| Cgrrf1 | -3.37 | 1.32E-05 | cell growth regulator with ring finger domain 1 |
| Pih1d1 | -3.64 | 1.08E-05 | PIH1 domain containing 1 |

**Supplementary Table 2:** List of abbreviations for gene symbols and gene names presented in the text and figures of the manuscript. The differentially expressed genes are typed in italics.

| **Symbol** | **Description** |
| --- | --- |
| Adcy1 | adenylate cyclase 1 |
| *Adcy2* | *adenylate cyclase 2* |
| *Adcy3* | *adenylate cyclase 3* |
| *Adcy4* | *adenylate cyclase 4* |
| *Adcy5* | *adenylate cyclase 5* |
| Adcy6 | adenylate cyclase 6 |
| Adcy7 | adenylate cyclase 7 |
| Adcy8 | adenylate cyclase 8 |
| Adcy9 | adenylate cyclase 9 |
| Akap5 | A kinase (PRKA) anchor protein 5 |
| Akt1 | thymoma viral proto-oncogene 1 |
| Akt2 | thymoma viral proto-oncogene 2 |
| *Akt3* | *thymoma viral proto-oncogene 3* |
| *Ap2a1* | *adaptor-related protein complex 2, alpha 1 subunit* |
| Ap2a2 | adaptor-related protein complex 2, alpha 2 subunit |
| Ap2b1 | adaptor-related protein complex 2, beta 1 subunit |
| *Ap2m1* | *adaptor-related protein complex 2, mu 1 subunit* |
| *Ap2s1* | *adaptor-related protein complex 2, sigma 1 subunit* |
| *Arhgef9* | *CDC42 guanine nucleotide exchange factor (GEF) 9* |
| Arntl | aryl hydrocarbon receptor nuclear translocator-like |
| Arrb2 | arrestin, beta 2 |
| *Atf2* | *activating transcription factor 2* |
| Atf4 | activating transcription factor 4 |
| Atf6b | activating transcription factor 6 beta |
| *Bcl2* | *B cell leukemia/lymphoma 2* |
| Braf | Braf transforming gene |
| Cacna1a | calcium channel, voltage-dependent, P/Q type, alpha 1A subunit |
| Cacna1b | calcium channel, voltage-dependent, N type, alpha 1B subunit |
| Cacna1c | calcium channel, voltage-dependent, L type, alpha 1C subunit |
| Cacna1d | calcium channel, voltage-dependent, L type, alpha 1D subunit |
| Cacna1f | calcium channel, voltage-dependent, alpha 1F subunit |
| Cacna1s | calcium channel, voltage-dependent, L type, alpha 1S subunit |
| Cacng2 | calcium channel, voltage-dependent, gamma subunit 2 |
| Cacng3 | calcium channel, voltage-dependent, gamma subunit 3 |
| Cacng4 | calcium channel, voltage-dependent, gamma subunit 4 |
| Calm1 | calmodulin 1 |
| Calm2 | calmodulin 2 |
| Calm3 | calmodulin 3 |
| *Calm4* | *calmodulin 4* |
| Calm5 | calmodulin 5 |
| Calml3 | calmodulin-like 3 |
| *Caly* | *calcyon neuron-specific vesicular protein* |
| *Camk2a* | *calcium/calmodulin-dependent protein kinase II alpha* |
| *Camk2b* | *calcium/calmodulin-dependent protein kinase II, beta* |
| Camk2d | calcium/calmodulin-dependent protein kinase II, delta |
| *Camk2g* | *calcium/calmodulin-dependent protein kinase II gamma* |
| Camk4 | calcium/calmodulin-dependent protein kinase IV |
| Chrm1 | cholinergic receptor, muscarinic 1, CNS |
| Chrm2 | cholinergic receptor, muscarinic 2, cardiac |
| *Chrm3* | *cholinergic receptor, muscarinic 3, cardiac* |
| *Chrm4* | *cholinergic receptor, muscarinic 4* |
| Chrm5 | cholinergic receptor, muscarinic 5 |
| Chrna2 | cholinergic receptor, nicotinic, alpha polypeptide 2 (neuronal) |
| Chrna3 | cholinergic receptor, nicotinic, alpha polypeptide 3 |
| Chrna4 | cholinergic receptor, nicotinic, alpha polypeptide 4 |
| Chrna5 | cholinergic receptor, nicotinic, alpha polypeptide 5 |
| *Chrna6* | *cholinergic receptor, nicotinic, alpha polypeptide 6* |
| Chrna7 | cholinergic receptor, nicotinic, alpha polypeptide 7 |
| Chrna9 | cholinergic receptor, nicotinic, alpha polypeptide 9 |
| Chrnb2 | cholinergic receptor, nicotinic, beta polypeptide 2 (neuronal) |
| *Chrnb3* | *cholinergic receptor, nicotinic, beta polypeptide 3* |
| Chrnb4 | cholinergic receptor, nicotinic, beta polypeptide 4 |
| Chrnd | cholinergic receptor, nicotinic, delta polypeptide |
| *Chrne* | *cholinergic receptor, nicotinic, epsilon polypeptide* |
| *Chrng* | *cholinergic receptor, nicotinic, gamma polypeptide* |
| Clock | circadian locomotor output cycles kaput |
| Creb1 | cAMP responsive element binding protein 1 |
| Creb3 | cAMP responsive element binding protein 3 |
| Creb3l1 | cAMP responsive element binding protein 3-like 1 |
| *Creb3l2* | *cAMP responsive element binding protein 3-like 2* |
| Creb3l3 | cAMP responsive element binding protein 3-like 3 |
| Creb3l4 | cAMP responsive element binding protein 3-like 4 |
| Creb5 | cAMP responsive element binding protein 5 |
| Dlg1 | discs, large homolog 1 (Drosophila) |
| Dlg3 | discs, large homolog 3 (Drosophila) |
| *Dlg4* | *discs, large homolog 4 (Drosophila)* |
| Drd1a | dopamine receptor D1A |
| Drd2 | dopamine receptor D2 |
| *Drd3* | *dopamine receptor D3* |
| *Drd4* | *dopamine receptor D4* |
| Drd5 | dopamine receptor D5 |
| *Epb4.1l1* | *erythrocyte protein band 4.1-like 1* |
| *Fos* | *FBJ osteosarcoma oncogene* |
| *Fyn* | *Fyn proto-oncogene* |
| *Gabarap* | *gamma-aminobutyric acid receptor associated protein* |
| *Gabarapl1* | *gamma-aminobutyric acid (GABA) A receptor-associated protein-like 1* |
| *Gabarapl2* | *gamma-aminobutyric acid (GABA) A receptor-associated protein-like 2* |
| *Gabbr1* | *gamma-aminobutyric acid (GABA) B receptor, 1* |
| Gabbr2 | gamma-aminobutyric acid (GABA) B receptor, 2 |
| Gabra1 | gamma-aminobutyric acid (GABA) A receptor, subunit alpha 1 |
| Gabra2 | gamma-aminobutyric acid (GABA) A receptor, subunit alpha 2 |
| *Gabra3* | *gamma-aminobutyric acid (GABA) A receptor, subunit alpha 3* |
| Gabra4 | gamma-aminobutyric acid (GABA) A receptor, subunit alpha 4 |
| Gabra5 | gamma-aminobutyric acid (GABA) A receptor, subunit alpha 5 |
| Gabra6 | gamma-aminobutyric acid (GABA) A receptor, subunit alpha 6 |
| *Gabrb1* | *gamma-aminobutyric acid (GABA) A receptor, subunit beta 1* |
| *Gabrb2* | *gamma-aminobutyric acid (GABA) A receptor, subunit beta 2* |
| *Gabrb3* | *gamma-aminobutyric acid (GABA) A receptor, subunit beta 3* |
| *Gabrd* | *gamma-aminobutyric acid (GABA) A receptor, subunit delta* |
| Gabre | gamma-aminobutyric acid (GABA) A receptor, subunit epsilon |
| Gabrg1 | gamma-aminobutyric acid (GABA) A receptor, subunit gamma 1 |
| *Gabrg2* | *gamma-aminobutyric acid (GABA) A receptor, subunit gamma 2* |
| Gabrg3 | gamma-aminobutyric acid (GABA) A receptor, subunit gamma 3 |
| *Gabrp* | *gamma-aminobutyric acid (GABA) A receptor, pi* |
| Gabrq | gamma-aminobutyric acid (GABA) A receptor, subunit theta |
| Gm15776 | predicted gene 15776 |
| Gm2436 | predicted gene 2436 |
| Gm2446 | predicted gene 2446 |
| Gm5741 | predicted gene 5741 |
| *Gna11* | *guanine nucleotide binding protein, alpha 11* |
| *Gnai1* | *guanine nucleotide binding protein (G protein), alpha inhibiting 1* |
| *Gnai2* | *guanine nucleotide binding protein (G protein), alpha inhibiting 2* |
| Gnai3 | guanine nucleotide binding protein (G protein), alpha inhibiting 3 |
| *Gnal* | *guanine nucleotide binding protein, alpha stimulating, olfactory type* |
| *Gnao1* | *guanine nucleotide binding protein, alpha O* |
| *Gnaq* | *guanine nucleotide binding protein, alpha q polypeptide* |
| Gnas | GNAS (guanine nucleotide binding protein, alpha stimulating) complex locus |
| Gnb1 | guanine nucleotide binding protein (G protein), beta 1 |
| *Gnb2* | *guanine nucleotide binding protein (G protein), beta 2* |
| Gnb3 | guanine nucleotide binding protein (G protein), beta 3 |
| Gnb4 | guanine nucleotide binding protein (G protein), beta 4 |
| Gnb5 | guanine nucleotide binding protein (G protein), beta 5 |
| Gng10 | guanine nucleotide binding protein (G protein), gamma 10 |
| Gng11 | guanine nucleotide binding protein (G protein), gamma 11 |
| Gng12 | guanine nucleotide binding protein (G protein), gamma 12 |
| Gng13 | guanine nucleotide binding protein (G protein), gamma 13 |
| *Gng2* | *guanine nucleotide binding protein (G protein), gamma 2* |
| *Gng3* | *guanine nucleotide binding protein (G protein), gamma 3* |
| Gng4 | guanine nucleotide binding protein (G protein), gamma 4 |
| Gng5 | guanine nucleotide binding protein (G protein), gamma 5 |
| Gng7 | guanine nucleotide binding protein (G protein), gamma 7 |
| Gng8 | guanine nucleotide binding protein (G protein), gamma 8 |
| Gngt1 | guanine nucleotide binding protein (G protein), gamma transducing activity polypeptide 1 |
| Gngt2 | guanine nucleotide binding protein (G protein), gamma transducing activity polypeptide 2 |
| Gphn | gephyrin |
| *Gria1* | *glutamate receptor, ionotropic, AMPA1 (alpha 1)* |
| *Gria2* | *glutamate receptor, ionotropic, AMPA2 (alpha 2)* |
| Gria3 | glutamate receptor, ionotropic, AMPA3 (alpha 3) |
| Gria4 | glutamate receptor, ionotropic, AMPA4 (alpha 4) |
| Grik3 | glutamate receptor, ionotropic, kainate 3 |
| *Grik4* | *glutamate receptor, ionotropic, kainate 4* |
| *Grin1* | *glutamate receptor, ionotropic, NMDA1 (zeta 1)* |
| Grin2a | glutamate receptor, ionotropic, NMDA2A (epsilon 1) |
| Grin2b | glutamate receptor, ionotropic, NMDA2B (epsilon 2) |
| Grin2c | glutamate receptor, ionotropic, NMDA2C (epsilon 3) |
| *Grin2d* | *glutamate receptor, ionotropic, NMDA2D (epsilon 4)* |
| Grip1 | glutamate receptor interacting protein 1 |
| *Gsk3a* | *glycogen synthase kinase 3 alpha* |
| *Gsk3b* | *glycogen synthase kinase 3 beta* |
| *Hap1* | *huntingtin-associated protein 1* |
| Hras1 | Harvey rat sarcoma virus oncogene 1 |
| Itpr1 | inositol 1,4,5-trisphosphate receptor 1 |
| Itpr2 | inositol 1,4,5-triphosphate receptor 2 |
| Itpr3 | inositol 1,4,5-triphosphate receptor 3 |
| Jak2 | Janus kinase 2 |
| *Kcnj10* | *potassium inwardly-rectifying channel, subfamily J, member 10* |
| *Kcnj12* | *potassium inwardly-rectifying channel, subfamily J, member 12* |
| Kcnj15 | potassium inwardly-rectifying channel, subfamily J, member 15 |
| Kcnj16 | potassium inwardly-rectifying channel, subfamily J, member 16 |
| Kcnj2 | potassium inwardly-rectifying channel, subfamily J, member 2 |
| Kcnj3 | potassium inwardly-rectifying channel, subfamily J, member 3 |
| Kcnj4 | potassium inwardly-rectifying channel, subfamily J, member 4 |
| Kcnj5 | potassium inwardly-rectifying channel, subfamily J, member 5 |
| Kcnj6 | potassium inwardly-rectifying channel, subfamily J, member 6 |
| Kcnj9 | potassium inwardly-rectifying channel, subfamily J, member 9 |
| *Kif5a* | *kinesin family member 5A* |
| *Kif5b* | *kinesin family member 5B* |
| *Kif5c* | *kinesin family member 5C* |
| Kras | v-Ki-ras2 Kirsten rat sarcoma viral oncogene homolog |
| LOC100047997 | cyclic AMP-dependent transcription factor ATF-2-like |
| LOC100048410 | guanine nucleotide-binding protein G(I)/G(S)/G(O) subunit gamma-5-like |
| Map2k1 | mitogen-activated protein kinase kinase 1 |
| Mapk1 | mitogen-activated protein kinase 1 |
| *Mapk10* | *mitogen-activated protein kinase 10* |
| Mapk11 | mitogen-activated protein kinase 11 |
| Mapk12 | mitogen-activated protein kinase 12 |
| *Mapk13* | *mitogen-activated protein kinase 13* |
| Mapk14 | mitogen-activated protein kinase 14 |
| Mapk3 | mitogen-activated protein kinase 3 |
| *Mapk8* | *mitogen-activated protein kinase 8* |
| *Mapk9* | *mitogen-activated protein kinase 9* |
| *Mdm2* | *transformed mouse 3T3 cell double minute 2* |
| Myo6 | myosin VI |
| Ncald | neurocalcin delta |
| Nras | neuroblastoma ras oncogene |
| *Nsf* | *N-ethylmaleimide sensitive fusion protein* |
| Pdpk1 | 3-phosphoinositide dependent protein kinase 1 |
| Pick1 | protein interacting with C kinase 1 |
| Pik3ca | phosphatidylinositol 3-kinase, catalytic, alpha polypeptide |
| Pik3cb | phosphatidylinositol 3-kinase, catalytic, beta polypeptide |
| Pik3cd | phosphatidylinositol 3-kinase catalytic delta polypeptide |
| Pik3cg | phosphoinositide-3-kinase, catalytic, gamma polypeptide |
| Pik3r1 | phosphatidylinositol 3-kinase, regulatory subunit, polypeptide 1 (p85 alpha) |
| Pik3r2 | phosphatidylinositol 3-kinase, regulatory subunit, polypeptide 2 (p85 beta) |
| *Pik3r3* | *phosphatidylinositol 3 kinase, regulatory subunit, polypeptide 3 (p55)* |
| *Pik3r5* | *phosphoinositide-3-kinase, regulatory subunit 5, p101* |
| Plcb1 | phospholipase C, beta 1 |
| *Plcb2* | *phospholipase C, beta 2* |
| Plcb3 | phospholipase C, beta 3 |
| Plcb4 | phospholipase C, beta 4 |
| Plcl1 | phospholipase C-like 1 |
| Ppp1ca | protein phosphatase 1, catalytic subunit, alpha isoform |
| *Ppp1cb* | *protein phosphatase 1, catalytic subunit, beta isoform* |
| Ppp1cc | protein phosphatase 1, catalytic subunit, gamma isoform |
| Ppp1r1b | protein phosphatase 1, regulatory (inhibitor) subunit 1B |
| Ppp2ca | protein phosphatase 2 (formerly 2A), catalytic subunit, alpha isoform |
| *Ppp2cb* | *protein phosphatase 2 (formerly 2A), catalytic subunit, beta isoform* |
| *Ppp2r1a* | *protein phosphatase 2 (formerly 2A), regulatory subunit A (PR 65), alpha isoform* |
| Ppp2r1b | protein phosphatase 2 (formerly 2A), regulatory subunit A (PR 65), beta isoform |
| Ppp2r2a | protein phosphatase 2 (formerly 2A), regulatory subunit B (PR 52), alpha isoform |
| Ppp2r2b | protein phosphatase 2 (formerly 2A), regulatory subunit B (PR 52), beta isoform |
| Ppp2r2c | protein phosphatase 2 (formerly 2A), regulatory subunit B (PR 52), gamma isoform |
| Ppp2r2d | protein phosphatase 2, regulatory subunit B, delta isoform |
| Ppp2r3a | protein phosphatase 2, regulatory subunit B'', alpha |
| Ppp2r3c | protein phosphatase 2, regulatory subunit B'', gamma |
| Ppp2r3d | protein phosphatase 2 (formerly 2A), regulatory subunit B'', delta |
| Ppp2r5a | protein phosphatase 2, regulatory subunit B (B56), alpha isoform |
| Ppp2r5b | protein phosphatase 2, regulatory subunit B (B56), beta isoform |
| Ppp2r5c | protein phosphatase 2, regulatory subunit B (B56), gamma isoform |
| Ppp2r5d | protein phosphatase 2, regulatory subunit B (B56), delta isoform |
| *Ppp2r5e* | *protein phosphatase 2, regulatory subunit B (B56), epsilon isoform* |
| *Ppp3ca* | *protein phosphatase 3, catalytic subunit, alpha isoform* |
| Ppp3cb | protein phosphatase 3, catalytic subunit, beta isoform |
| Ppp3cc | protein phosphatase 3, catalytic subunit, gamma isoform |
| *Prkaca* | *protein kinase, cAMP dependent, catalytic, alpha* |
| *Prkacb* | *protein kinase, cAMP dependent, catalytic, beta* |
| *Prkca* | *protein kinase C, alpha* |
| Prkcb | protein kinase C, beta |
| Prkcg | protein kinase C, gamma |
| Prkx | protein kinase, X-linked |
| Raf1 | v-raf-leukemia viral oncogene 1 |
| *Rasgrf1* | *RAS protein-specific guanine nucleotide-releasing factor 1* |
| Rps6ka2 | ribosomal protein S6 kinase, polypeptide 2 |
| *Rps6ka3* | *ribosomal protein S6 kinase polypeptide 3* |
| Rps6ka6 | ribosomal protein S6 kinase polypeptide 6 |
| Rras | Harvey rat sarcoma oncogene, subgroup R |
| Scn1a | sodium channel, voltage-gated, type I, alpha |
| Slc6a3 | solute carrier family 6 (neurotransmitter transporter, dopamine), member 3 |
| Th | tyrosine hydroxylase |
| Trak2 | trafficking protein, kinesin binding 2 |
